# Identification of herbal teas and their compounds eliciting antiviral activity against SARS-CoV-2 in vitro

**DOI:** 10.1186/s12915-022-01468-z

**Published:** 2022-11-30

**Authors:** Vu Thuy Khanh Le-Trilling, Denise Mennerich, Corinna Schuler, Roman Sakson, Julia K. Lill, Siva Swapna Kasarla, Dominik Kopczynski, Stefan Loroch, Yulia Flores-Martinez, Benjamin Katschinski, Kerstin Wohlgemuth, Matthias Gunzer, Folker Meyer, Prasad Phapale, Ulf Dittmer, Albert Sickmann, Mirko Trilling

**Affiliations:** 1 Institute for Virology, University Hospital Essen, University of Duisburg-Essen, Virchowstr. 179, 45147 Essen, Germany; 2 Leibniz-Institut Für Analytische Wissenschaften - ISAS - E.V., Dortmund, Germany; 3 Institute for Experimental Immunology and Imaging, University Hospital Essen, University of Duisburg-Essen, Essen, Germany; 4 Institute for AI in Medicine, University Hospital Essen, University of Duisburg-Essen, Essen, Germany; 5 Medizinische Fakultät, Ruhr-Universität Bochum, Bochum, Germany; 6 Department of Chemistry, College of Physical Sciences, University of Aberdeen, Aberdeen, UK

**Keywords:** COVID-19, SARS-CoV-2, Antiviral, Herbal, Perilla, Sage, Mint, Thyme, Heme oxygenase 1, Proteome

## Abstract

**Background:**

The SARS-CoV-2/COVID-19 pandemic has inflicted medical and socioeconomic havoc, and despite the current availability of vaccines and broad implementation of vaccination programs, more easily accessible and cost-effective acute treatment options preventing morbidity and mortality are urgently needed. Herbal teas have historically and recurrently been applied as self-medication for prophylaxis, therapy, and symptom alleviation in diverse diseases, including those caused by respiratory viruses, and have provided sources of natural products as basis for the development of therapeutic agents. To identify affordable, ubiquitously available, and effective treatments, we tested herbs consumed worldwide as herbal teas regarding their antiviral activity against SARS-CoV-2.

**Results:**

Aqueous infusions prepared by boiling leaves of the *Lamiaceae* perilla and sage elicit potent and sustained antiviral activity against SARS-CoV-2 when applied after infection as well as prior to infection of cells. The herbal infusions exerted in vitro antiviral effects comparable to interferon-β and remdesivir but outperformed convalescent sera and interferon-α2 upon short-term treatment early after infection. Based on protein fractionation analyses, we identified caffeic acid, perilla aldehyde, and perillyl alcohol as antiviral compounds. Global mass spectrometry (MS) analyses performed comparatively in two different cell culture infection models revealed changes of the proteome upon treatment with herbal infusions and provided insights into the mode of action. As inferred by the MS data, induction of heme oxygenase 1 (HMOX-1) was confirmed as effector mechanism by the antiviral activity of the HMOX-1-inducing compounds sulforaphane and fraxetin.

**Conclusions:**

In conclusion, herbal teas based on perilla and sage exhibit antiviral activity against SARS-CoV-2 including variants of concern such as Alpha, Beta, Delta, and Omicron, and we identified HMOX-1 as potential therapeutic target. Given that perilla and sage have been suggested as treatment options for various diseases, our dataset may constitute a valuable resource also for future research beyond virology.

**Supplementary Information:**

The online version contains supplementary material available at 10.1186/s12915-022-01468-z.

## Background

Fossil records suggest that humans may have applied plants as medicine at least from the Middle Paleolithic age some 60,000 years ago [1, 2]. Across cultures and spanning thousands of years, humans consumed aqueous plant infusions as teas. The first textual reference to tea consumption dates back to 59 years before the current era (BCE) and physical evidence even dates back to 255 ± 80 years BCE [3]. In addition to reasons of enjoyment and taste, teas are frequently applied for disease prophylaxis, therapy, or symptom alleviation. A major distinction is made between genuine teas based on *Camellia sinensis* infusions versus various types of herbal teas. For the latter, parts of other plants are boiled in water generating complex aqueous infusions. Especially members of the *Lamiaceae* family comprising sage (*Salvia officinalis*) and perilla (*Perilla frutescens)* are ubiquitously used to prepare herbal teas. Additionally, important spices such as thyme, mint, basil, rosemary, marjoram, oregano, and lavender also belong to the *Lamiaceae*. Across the world, the edible plant perilla and its variations have a variety of names such as *Tía tô* (Vietnam), *rattlesnake weed* (US), *silam* (India and Nepal), *shiso* and *egoma* (Japan), *deulkkae* (Korea), and *zĭsū* and *sūzǐ* (China) highlighting its broad distribution. Members of the *Lamiaceae* family are well described for their medicinal effects against various diseases including pneumonia and cough [4]. While perilla is very popular in Asia, the related plant sage is more common in Europe and America. Intriguingly, perilla and sage extracts indeed possess antimicrobial activities (e.g.[5, 6],). In the era of modern medicine, some people have reservations concerning the use of traditional and herbal medicines. However, a highly relevant fraction of recently approved modern therapeutics directly or indirectly originates from natural products [7]. In this respect, the antimalarial lactone artemisinin derived from the sweet wormwood (*Artemisia annua*) is among the best-known examples [8, 9].

Humans have been exposed to coronaviruses (CoV) for ages, given their broad prevalence in mammals (e.g., bats) and birds. At least seven CoVs are capable of autochthonous propagation in the human population: human CoV (HCoV)-HKU1, HCoV-NL63, HCoV-229E, HCoV-OC43, HCoV-SARS, HCoV-MERS, and the severe acute respiratory syndrome coronavirus 2 (SARS-CoV-2). The latter causes the current global pandemic of coronavirus disease 2019 (COVID-19). SARS-CoV-2 was first recognized and genetically defined in Wuhan, China [10, 11]. In various aspects, it shows similarities to SARS-CoV-1; however, it also exhibits certain specialties [12, 13] such as the capacity of very efficient replication in the upper respiratory tract and the corresponding efficacy of human-to-human transmission. Given the broad coverage of this topic, we refer the reader to review articles concerning SARS-CoV-2 and COVID-19 (e.g.[14–18],). At the time of writing, more than 628 million individuals experienced laboratory-confirmed SARS-CoV-2 infections and over 6.57 million people succumbed in the context of COVID-19. According to the Johns Hopkins dashboard [19], all relevant countries and regions are affected by SARS-CoV-2 cases—several of which are developing nations with very limited resources for the medical sector, especially when faced with overwhelming numbers of infected individuals [20]. Since the onset of the COVID-19 pandemic in December 2019, SARS-CoV-2 has continuously evolved, with numerous variants emerging across the world. Depending on prevalence and clinical and epidemiological characteristics, these variants are classified as “variant of interest” (VOI), “variant under monitoring” (VUM), and “variant of concern” (VOC). As of March 2022, there are five SARS-CoV-2 lineages designated as VOCs (Alpha, Beta, Gamma, Delta, and Omicron variants). Delta was the most prevalent variant in 2021, but in 2022, Omicron has overtaken Delta as the predominant variant (https://nextstrain.org/ncov/gisaid/global, [21]). Certain VOCs exhibit increased transmissibility compared to the original virus and others the potential to increase disease severity. Moreover, some VOCs show decreased susceptibility to vaccine- and infection-induced immune responses, and thus possess an elevated ability to re-infect previously infected and recovered as well as vaccinated individuals.

Based on the facts that CoVs are present in animals such as rodents, bats, pigs, and cats residing in utmost proximity of human settlements and civilization and seem to have caused human epidemics in the past [22, 23], we speculated that human culture might provide certain behavioral adaptations to coronavirus infections. Such knowledge may be applicable to alleviate some of the hardship and suffering caused by SARS-CoV-2 in a process of “cultural repurposing”. People with respiratory diseases often consume herbal products and teas in attempts of self-medication. Faced with the COVID-19 pandemic, people reported that they have changed their behavior accordingly. In two studies comprising thousands of people, up to 57.6% of individuals reported having used nutritional supplements or herbal products, usually as teas, in attempts to protect themselves from COVID-19 [24, 25]. Therefore, we wondered how effective herbal teas actually are against SARS-CoV-2 and tested the in vitro antiviral activity of herbal infusions against SARS-CoV-2 variants including VOCs.

## Results

### Perilla and sage contain water-soluble heat-stable components active against SARS-CoV-2 replication

**Fig. 1:**
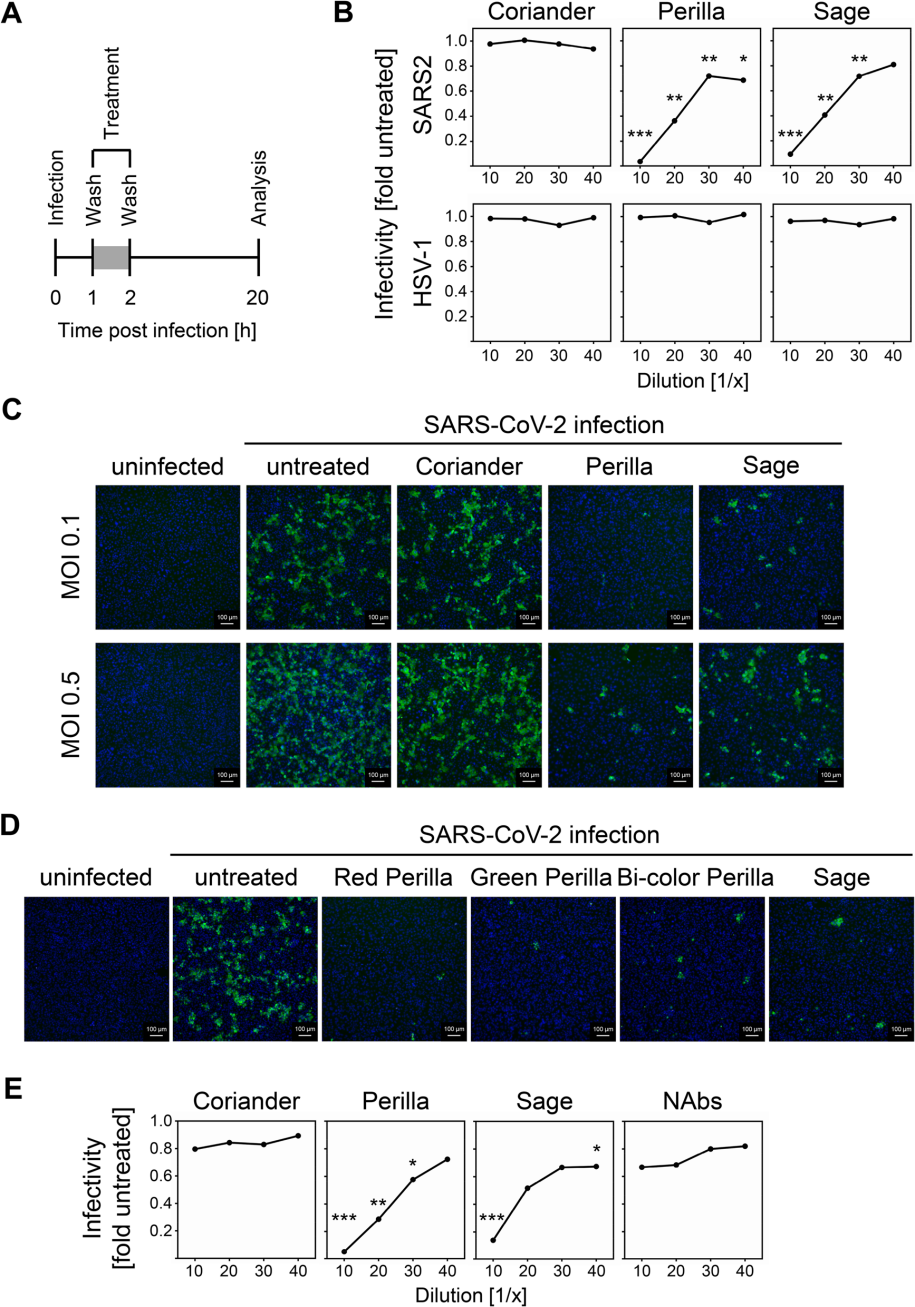
Perilla and sage contain water-soluble heat-stable components active against SARS-CoV-2 in vitro replication. **A** Scheme of the experimental setup for the in vitro analysis of therapeutic effects against SARS-CoV-2. **B** Representative dose-response curves of SARS-CoV-2-infected Vero E6 cells (2000 PFU per well) after treatment with aqueous infusions of coriander, perilla, or sage. Upper panel shows the effect on SARS-CoV-2 replication. Lower panel depicts the effect on HSV-1 replication. SARS-CoV-2 replication was analyzed at 20 h p.i. by icELISA, HSV-1:GFP replication was determined at 48 h p.i. by quantification of fluorescence. Data are expressed as relative change in infectivity compared to the untreated control. Each condition was analyzed in triplicate. See Additional file 1 for individual data values and “Methods” section and Additional file 2: Fig. S1 for details. The comparison of the herb-treated samples of SARS-CoV-2 to the untreated controls by one-way ANOVA showed for all dilutions of coriander no significance and for all dilutions of perilla and sage significance. The perilla- and sage-treated conditions of SARS-CoV-2 were also compared to the corresponding coriander-treated condition (same dilution) and these results are depicted in the diagram. *, *p* < 0.05. **, *p* < 0.01. ***, *p* < 0.001. The comparison of the herb-treated samples of HSV-1 to the untreated controls showed for all dilutions of all tested herbs no significance. **C**, **D** Visualization of SARS-CoV-2 infection upon treatment with herbal infusions. Vero E6 cells were infected (MOI 0.1) and treated (1/10 dilution) as shown in **A**. α-S mAb and a Cy2-coupled secondary antibody were used for immunofluorescence staining (green). Nuclei were counterstained with DAPI (blue). **E** Representative dose-response curves of SARS-CoV-2-infected Vero E6 cells (2000 PFU per well) after treatment (as shown in **A**) with aqueous infusions of coriander, perilla, sage, or SARS-CoV-2 convalescent serum (serum 6 from[26]. with mid-high 50% neutralization titer of 256). Each condition was analyzed in triplicate. See Additional file 1 for individual data values. The perilla- and sage-treated conditions were compared to the corresponding NAbs-treated condition (same dilution) by one-way ANOVA. **, *p* < 0.01. ***, *p* < 0.001

To evaluate two universally available *Lamiaceae* commonly used in traditional medicine, perilla and sage, in terms of their ability to elicit antiviral activity against SARS-CoV-2, we applied a cell culture experimental setup that reflects short-term incubation of infected cells with herbal teas. We infected Vero E6 cells, a highly SARS-CoV-2-permissive cell line derived from African green monkey and broadly used for SARS-CoV-2 infections, for 1 h before the virus suspension was removed and different dilutions of aqueous infusions of perilla and sage were added afterwards (Fig. 1A). As control, we included coriander, an herb that does not belong to the family of *Lamiaceae* and that is to our knowledge not commonly used as medicinal herb. We applied aqueous infusions that were prepared by boiling up the coriander, perilla, and sage leaves and subsequent simmering at 60 °C for 2 h to ensure efficient extraction of water-soluble components. Intriguingly, the short-term treatment with perilla and sage infusions significantly inhibited the replication of SARS-CoV-2 (Fig. 1B, upper panel; see Additional file 1 for individual data values and “Methods” section and Additional file 2: Fig. S1 for details concerning the calculation of the infectivity). This effect did not appear to constitute a general antiviral activity of the infusions, since HSV-1 replication in treated Vero E6 cells was not inhibited (Fig. 1B, lower panel). To visualize the impact of the herbal teas on the SARS-CoV-2 replication, we repeated the experiment with two different doses of virus and stained the infected cells for immunofluorescence microscopy. As depicted in Fig. 1C, the number of infected cells (green) was clearly diminished after treatment with perilla and sage infusions. The antiviral activity was still evident even when a high amount of virus was used for infection (0.5 PFU/cell). When we evaluated different members of the family of *Perilla frutescens* (red perilla, green perilla, and bi-color perilla), we observed antiviral activity in all three cases (Fig. 1D). No comparable antiviral activity was observed under the aforementioned conditions when ginger, fennel, chamomile flower, and Greek mountain teas were tested (data not shown). To confirm that the experimental setup allows reporting of antiviral activity affecting post-entry steps, we included a SARS-CoV-2 convalescent serum sample with shown neutralizing capacity [26] in our analysis. We observed that the perilla and sage infusions outperformed the effect of the convalescent serum (Fig. 1E; NAbs, neutralizing antibodies) under these experimental conditions, suggesting that the herbs perilla and sage contain components active against SARS-CoV-2 replication by interfering with a post-entry step, which is resistant to entry inhibitors such as NAbs. Since the components were extracted by boiling herbs in water, we concluded that the antiviral activity is elicited by water-soluble heat-stable compound(s). When we used a second clinical SARS-CoV-2 isolate to test the susceptibility towards the herbal components, we observed almost identical dose responses (Additional file 3: Fig. S2). The combined results of several independent experiments (using two distinct SARS-CoV-2 isolates, B.1 and B.1.1.232, for the infection of Vero E6 and α-S or α-N antibodies for staining) revealed highly significant antiviral activity of all tested dilutions of the perilla infusion as well as of the 1/10 and 1/20 dilutions of the sage infusion (Additional file 3: Fig. S2). Cell viability was determined to exclude cytotoxicity as reason for diminished viral replication (Additional file 4: Fig. S3A). In conclusion, the edible *Lamiaceae* plants perilla and sage contain water-soluble heat-stable components that exhibit potent antiviral activity against SARS-CoV-2 in cell culture.

### Preserved sage and perilla leaves retain bioactive compounds

Per liter, our herbal infusions were prepared from 150 g of fresh herbal material corresponding to 15–30 g of dried herbal material (assuming a water content of 80–90% in fresh herbs). Accordingly, the 1/20 dilutions of these infusions used in our experiments are less concentrated than infusions typically consumed as teas (the industry standard being 3 g tea per 250 ml cup), e.g., from standard tea bags (1–3 g of tea intended for one cup of tea). To further adapt the experimental setup to realistic conditions of tea preparation, we shortened the treatment time from 1 h to 30 min. Even under this condition, significant reduction of viral replication was observed (Additional file 4: Fig. S3B). Next, we compared the antiviral activity of infusions prepared from fresh or dried herb leaves. As we showed (Additional file 4: Fig. S3C), dried sage leaves retained most of the antiviral component(s) whereas dried perilla was less effective as compared to fresh perilla leaves although significant inhibition of viral replication was still observed for the 1/10 dilutions. To assess whether conservation of perilla by freezing could be superior to drying in terms of preserving the antiviral component(s), we first tested if the antiviral activity of perilla infusions is reduced by freeze–thaw cycles. Since this did not seem to be the case (Additional file 4: Fig. S3D), infusions prepared from fresh and frozen perilla leaves were compared. The comparison revealed that the preservation of the herbs by freezing was preferable to drying (Additional file 4: Fig. S3E). The fact that perilla loses parts of its antiviral potency is compatible with the conclusion that at least one of its antiviral constituents may be volatile (see below). Since the herbal infusions were prepared by boiling, simmering, and overnight incubation (see “Methods” section), we also tested if the standard procedure of herbal tea preparation using dried sage leaves is sufficient to extract the antiviral component(s). To this end, dried sage leaves were boiled up in water and incubated for 10 min before the herb leaves were removed. When this 10-min infusion of dried sage was compared to the overnight infusion, very similar dose–response curves were observed (Additional file 4: Fig. S3F).

### Perilla and sage elicit antiviral activity when applied prior to in vitro infections

Having observed potent antiviral activity of perilla and sage after only 1 h of treatment, we wondered whether the herbs might also elicit antiviral activity after pre-treatment of cells. Therefore, Vero E6 cells were treated 1 h prior to infection with different dilutions of the herbal infusions before the supernatant including the herbal components was removed. Subsequently, SARS-CoV-2 infection was performed and the virus suspension was replaced by fresh medium at 1 h p.i. (Additional file 5: Fig. S4A). By removing the herbal infusions before infection, we aimed to primarily assess prophylactic antiviral effects based on cellular responses rather than direct virucidal elimination of infectious virus particles. The analysis of combined results of 6 independent experiments using two distinct SARS-CoV-2 isolates and α-S or α-N antibodies for staining showed highly significant decrease of infectivity, especially upon pre-treatment with the perilla infusion (Additional file 5: Fig. S4B). To compare the extent of treatment and pre-treatment antiviral capacity, we conducted an experiment in which we treated and pre-treated the infected cells in parallel. As already indicated by the results of the independent experiments (Additional file 3: Fig. S2A-B and Additional file 5: Fig. S4B), postinfection treatment elicited stronger antiviral activity for both perilla and sage (Additional file 5: Fig. S4C-E).

### Perilla and sage confer protection against SARS-CoV-2 infection in human cells

**Fig. 2:**
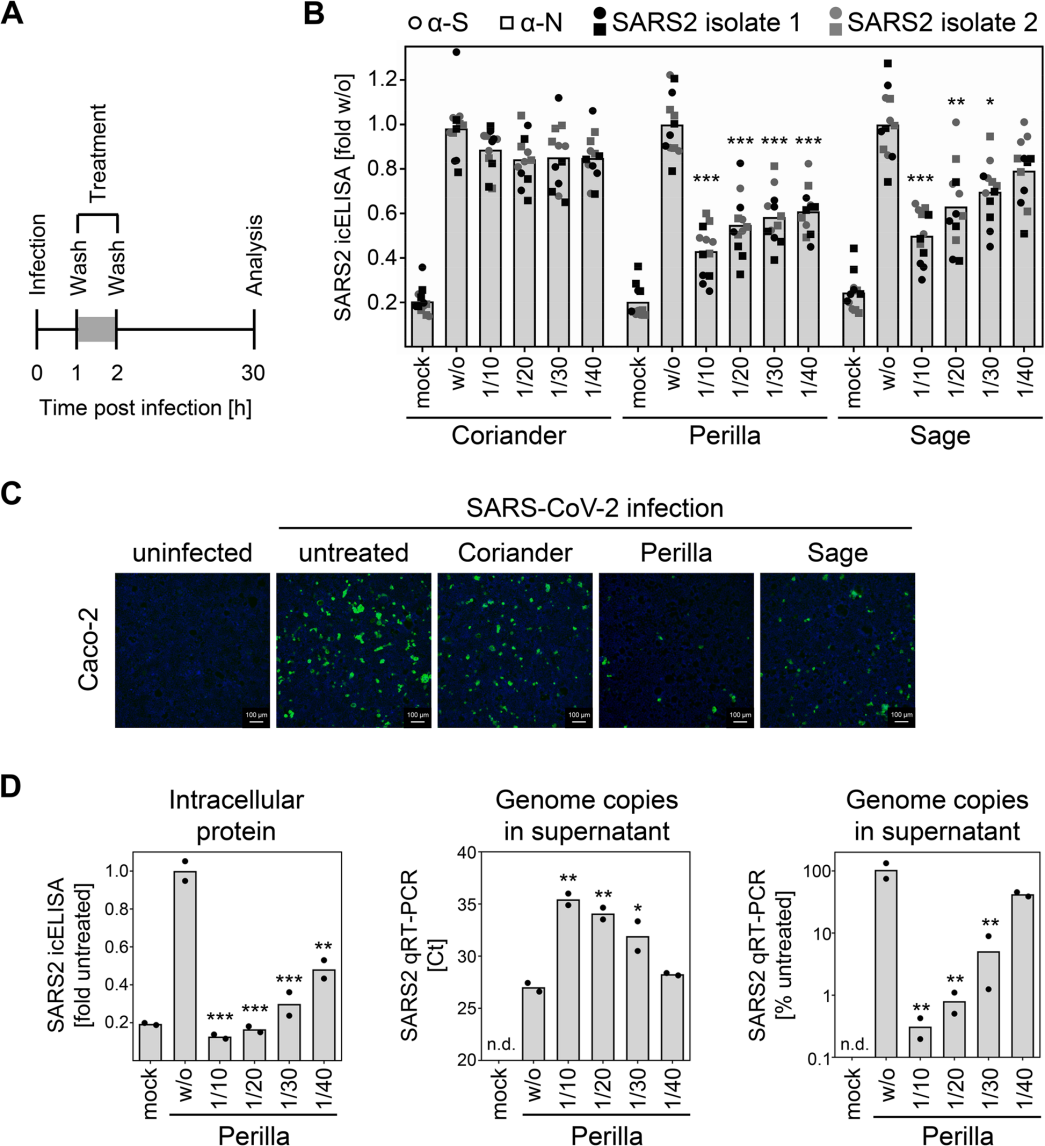
Perilla and sage confer protection against SARS-CoV-2 infection in human cells. **A** Scheme of the experimental setup for the in vitro analysis of antiviral activity against SARS-CoV-2 in human Caco-2 cells. **B** Pooled icELISA data of 4 independent experiments using two distinct SARS-CoV-2 isolates (B.1. and B1.1.232) for infection of Caco-2 cells and α-S or α-N mAbs for staining. Data are expressed as relative change in optical density compared to the untreated control. The perilla- and sage-treated conditions were compared to the corresponding coriander-treated condition (same dilution) by one-way ANOVA. *, *p* < 0.05. **, *p* < 0.01. ***, *p* < 0.001. **C** Visualization of SARS-CoV-2 infection upon treatment with herbal infusions. Human Caco-2 cells were infected and treated as shown in **A**. α-S mAb and a Cy2-coupled secondary antibody were used for immunofluorescence staining (green). Nuclei were counterstained with DAPI (blue). **D** Human Caco-2 cells were infected and treated as shown in Additional file 5: Fig. S5A. At 30 h p.i., supernatant was collected for RNA preparation and subsequent qRT-PCR analysis. Cells were fixed and analyzed by icELISA using α-S mAb for staining. icELISA data are expressed as relative change in optical density compared to the untreated control. qRT-PCR data are shown in Ct value and calculated relative change in genome copies compared to the untreated control. Each condition was analyzed in duplicate. See Additional file 1 for individual data values. The perilla-treated conditions were compared to the untreated control by one-way ANOVA. *, *p* < 0.05. **, *p* < 0.01. ***, *p* < 0.001

Since cells differ concerning the mode of entry of SARS-CoV-2 [27], a second independent cell line was tested. Caco-2, a human SARS-CoV-2-permissive cell line, were used to analyze the antiviral activity elicited by perilla and sage infusions in human cells. We have observed that SARS-CoV-2 replication is more protracted in Caco-2 cells compared to Vero E6 cells [26]. Therefore, the experimental setup was adapted by increasing the time of treatment as well as the time of infection before analysis (Additional file 6: Fig. S5A). We observed a strong decrease in infectivity in perilla- and sage-treated Caco-2 cells (Additional file 6: Fig. S5B). Encouraged by this result, we applied the same treatment regimen of 1 h as was used for Vero E6 cells (Fig. 2A). This early short-term treatment potently inhibited SARS-CoV-2 replication, even in cells with a protracted viral replication cycle (Fig. 2B). To visualize the antiviral activity, Caco-2 cells were infected with and without treatment (as depicted in Fig. 2A) and were fixed for fluorescence microscopic analysis. This analysis showed clearly visible differences in the number of Spike-positive cells (Fig. 2C). To corroborate the data obtained so far, we analyzed the antiviral activity of herbal infusions by staining the intracellular spike protein by in-cell-ELISA (icELISA [26]) as well as quantifying the viral genomes in the supernatant by qRT-PCR. The results showed that the icELISA data reflected the decrease in viral replication and the resulting decline in viral progeny (Fig. 2D).

### The herbal infusions exert in vitro antiviral effects comparable to IFNβ and remdesivir and outperform IFNα2 upon short-term treatment

**Fig. 3:**
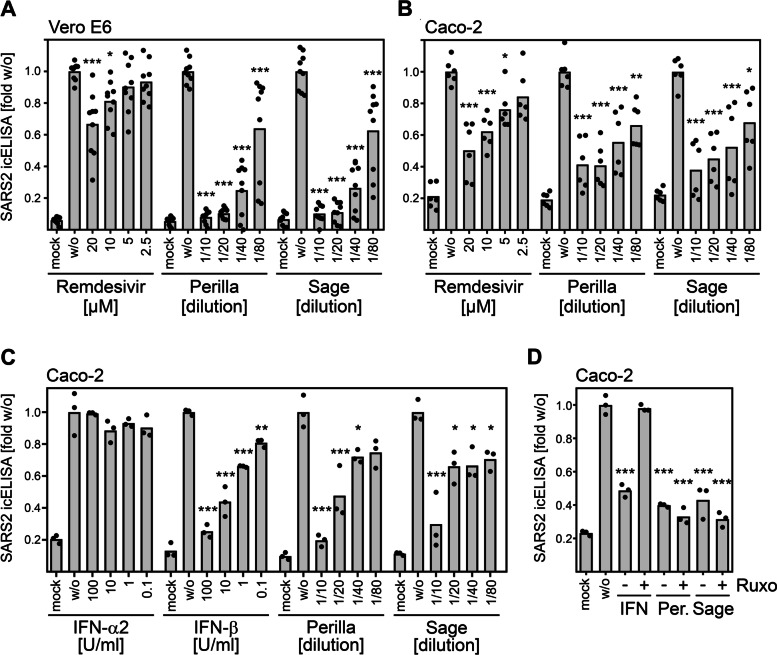
The herbal infusions exerted comparable antiviral effects to IFNβ and remdesivir and outperformed IFNα2 upon short-term treatment. **A, B** Pooled icELISA data of SARS-CoV-2-infected cells (**A**, Vero E6, 3 independent experiments; **B**, Caco-2, 2 independent experiments) after treatment with herbal infusions or remdesivir at 1 h p.i. for 1 h. Vero E6 and Caco-2 were fixed at 24 h p.i. and stained with α-S and α-N, respectively. Data are expressed as relative change in optical density compared to the untreated control. The treated conditions were compared to the untreated control by one-way ANOVA. *, *p* < 0.05. **, *p* < 0.01. ***, *p* < 0.001. **C** icELISA data of SARS-CoV-2-infected Caco-2 cells after treatment with herbal infusions or indicated interferons at 1 h p.i. for 1.5 h. Cells were fixed at 30 h p.i. and stained with α-S mAb. Each condition was analyzed in triplicate. See Additional file 1 for individual data values. The treated conditions were compared to the untreated control by one-way ANOVA. *, *p* < 0.05. **, *p* < 0.01. ***, *p* < 0.001. **D** icELISA data of SARS-CoV-2-infected Caco-2 cells after treatment with IFNβ or herbal infusions (P, perilla; S, sage) at 1 h p.i. for 1.5 h in the absence or presence of the Janus kinase inhibitor ruxolitinib (4 µM). See Additional file 1 for individual data values. The treated conditions were compared to the untreated control by one-way ANOVA. ***, *p* < 0.001

Interferons mediate innate immunity against viruses, are discussed as treatment option for COVID-19, and constitute benchmarks for antiviral activities. Another antiviral drug authorized for emergency use in COVID-19 patients is remdesivir, an adenosine nucleotide analog prodrug inhibiting the viral RNA-dependent RNA polymerase. To assess the potency of the herbal infusions, we compared treatment with perilla and sage infusions with treatment with remdesivir, IFNα2, and IFNβ in vitro. We infected Vero E6 or Caco-2 cells with SARS-CoV-2 and treated the infected cells at 1 h postinfection for 1 h before the treatment was removed. The antiviral activity was determined by quantification of viral proteins by icELISA. Under herein used experimental condition, perilla and sage infusions showed higher antiviral potency than remdesivir in Vero E6 cells (Fig. 3A) and comparable antiviral activity in infected Caco-2 cells (Fig. 3B). As expected, extended long-term remdesivir treatment showed potent antiviral activity, confirming its integrity (Additional file 7: Fig. S6). When the herbal infusions were compared to IFNs, we observed similar effects to short-term IFNβ treatment but higher potency than short-term treatment with IFNα2 (Fig. 3C). Vero cells harbor deletions of all genes coding for IFNα and IFNβ [28, 29]. Thus, the herbal infusions elicited antiviral activity in cells lacking the ability to generate IFNα/β. Since other antiviral IFNs such as type III IFNs (IFNλ) exist, we tested if herbal infusions rely on IFNs for their antiviral activity by applying ruxolitinib. The janus kinase inhibitor ruxolitinib prevents IFN signaling along the Jak-STAT pathway and precludes that IFNs elicit antiviral activity (see, e.g.[30],). Consistent with the notion that the herbal infusions act in a different manner than the IFNs, treatment with the Janus kinase inhibitor ruxolitinib abrogated the antiviral activity of IFNβ but not perilla and sage (Fig. 3D).

### Identification of antiviral components following size exclusion and protein fractionation analyses

**Fig. 4:**
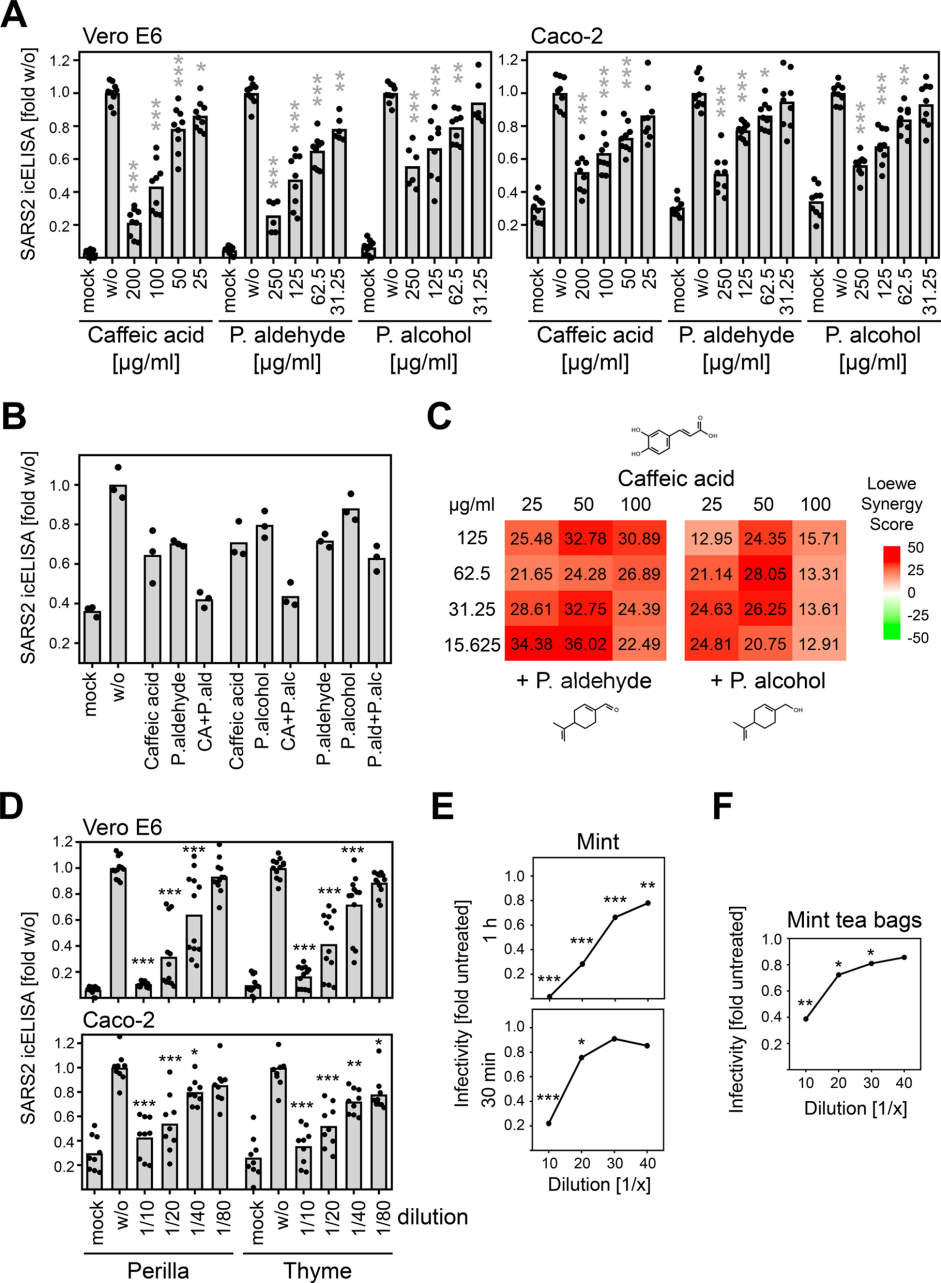
Synergistic antiviral activity of caffeic acid, perilla aldehyde, and perillyl alcohol. **A** Pooled icELISA data of 3 independent experiments of SARS-CoV-2-infected Vero E6 and Caco-2 cells after treatment with components of herbal infusions at 1 h p.i or 1.5 h p.i. for 1.5 h. Cells were fixed at 20 to 24 h p.i. and stained with α-S (Vero E6) or α-N mAb (Caco-2). Each condition was analyzed in triplicate. The treated conditions were compared to the untreated control by one-way ANOVA. *, *p* < 0.05. **, *p* < 0.01. ***, *p* < 0.001. IC50 values (compound concentration sufficient to reduce the virus-specific icELISA signal to 50%) were calculated by nonlinear regression for Vero E6 and Caco-2. Caffeic acid, 82.95 and 34.64 µg/ml. Perilla aldehyde, 94.99 and 77.04 µg/ml. Perillyl alcohol, 252.7 and 66.62 µg/ml. **B** icELISA data of SARS-CoV-2-infected Vero E6 cells after treatment with indicated compounds (caffeic acid, 25 µg/ml; perilla aldehyde and perillyl alcohol, 125 µg/ml) at 1 h p.i. for 1.5 h. Cells were fixed at 20 h p.i. and stained with α-S mAb. Each condition was analyzed in triplicate. See Additional file 1 for individual data values. **C** Combination of different concentrations of caffeic acid and perilla aldehyde or perillyl alcohol were tested for their antiviral activity. The inhibition (%) compared to the untreated control was calculated and the mean values of 3 replicates were included to test for synergistic effects. The R package SynergyFinder was used to calculate the Loewe synergy score [33]. The analysis was performed without impute method and baseline correction. **D** Pooled icELISA data of SARS-CoV-2-infected cells (Vero E6, 4 independent experiments; Caco-2, 3 independent experiments) after treatment with perilla or thyme infusion. Data are expressed as relative change in optical density compared to the untreated control. All treated conditions were compared to the untreated control by one-way ANOVA. *, *p* < 0.05. **, *p* < 0.01. ***, *p* < 0.001. **E, F** Dose-response curves of SARS-CoV-2-infected Vero E6 cells (2000 PFU per well) after treatment for 1 h or 30 min with aqueous infusions of mint (**E**, fresh mint; **F**, 1 h treatment with aqueous infusion of 2 commercially available mint tea bags in 100 ml water). Each condition was analyzed in triplicate. See Additional file 1 for individual data values. The mint-treated conditions were compared to the untreated control by one-way ANOVA. *, *p* < 0.05. **, *p* < 0.01. ***, *p* < 0.001

Since the herbal infusions contain a large number of different components, it would have been difficult to determine the active substances without additional information regarding the nature of these active components. To narrow down candidate substances, we fractionated the components of the herbal infusions according to their molecular size and tested the antiviral activity of these fractions. When we tested the fractions obtained by use of Amicon 100 K, 30 K, and 10 K filters, we were able to assign the active component(s) to substances less than 10 kDa in size (Additional file 8: Fig. S7). To further reduce the number of potential candidates, we used Amicon 10 K and 3 K filters for fractionation. Additionally, we prepared by dialysis the fraction of substances larger than 1 kDa. The analysis of these fractions revealed that the dialyzed herbal infusions exhibited decreased antiviral activity indicating that components less than 1 kDa in size contribute to the antiviral activity of perilla (Additional file 9: Fig. S8A) and sage infusions (Additional file 9: Fig. S8B). In light of these findings, we reviewed the literature for candidate substances. Based on published lists of perilla components (e.g.[31, 32],), we tested various compounds present in *Lamiaceae*. We did not detect antiviral activity against SARS-CoV-2 when we tested apigenin, luteolin, rosmarinic acid, and tormentic acid (data not shown). Interestingly, when we analyzed cinnamic acid, hydroxy-cinnamic acid, and dihydroxy-cinnamic acid, only the treatment with dihydroxy-cinnamic acid diminished SARS-CoV-2 replication in Vero E6 (data not shown and Fig. 4A) and Caco-2 cells (Additional file 10: Fig. S9A, Fig. 4A). This finding is remarkable, since hydroxy-cinnamic acid and dihydroxy-cinnamic acid (that is more familiar under the name caffeic acid) differ only by a single hydroxyl group. In addition, we found that, besides caffeic acid, perilla aldehyde and perillyl alcohol also exhibit antiviral activity against SARS-CoV-2 (Additional file 10: Fig. S9B, Fig. 4A). When we combined the compounds, we observed an increase in antiviral activity (Fig. 4B). These results suggest that the effect of the herbal infusions is based on multiple components that presumably act through additive or even synergistic mechanisms. To test for synergy, we combined graded concentrations of caffeic acid with increasing doses of perilla aldehyde or perillyl alcohol and compared the effect on SARS-CoV-2. Based on high Loewe synergy scores ranging from 12.91 to 36.02 (Fig. 4C), the compounds were found to act synergistically.

To evaluate whether knowledge of the active components could help predict additional antiviral herbs, we searched for plants containing high amounts of caffeic acid. Thyme and mint are herbs containing high caffeic acid concentrations (http://phenol-explorer.eu/contents/polyphenol/457). Therefore, we analyzed aqueous infusions of thyme and mint. We observed potent antiviral activity of both herbs against SARS-CoV-2 in Vero E6 and Caco-2 cells (Fig. 4D,E). Mint showed in experimental pre-treatment as well as treatment regimens antiviral activity, and the antiviral principle was resistant to freeze–thaw cycles (Additional file 11: Fig. S10). Thus, the identification of antiviral compounds has predictive value for the identification of additional antiviral herbs, and may pave the way to novel therapeutics for COVID-19. Intriguingly, the content of commercially available mint tea bags elicited potent antiviral activity in cell culture (Fig. 4F).

### Global mass spectrometry (MS) analyses proposed induction of HMOX-1 as anti-SARS-CoV-2 effector mechanism of herbal infusions

**Fig. 5:**
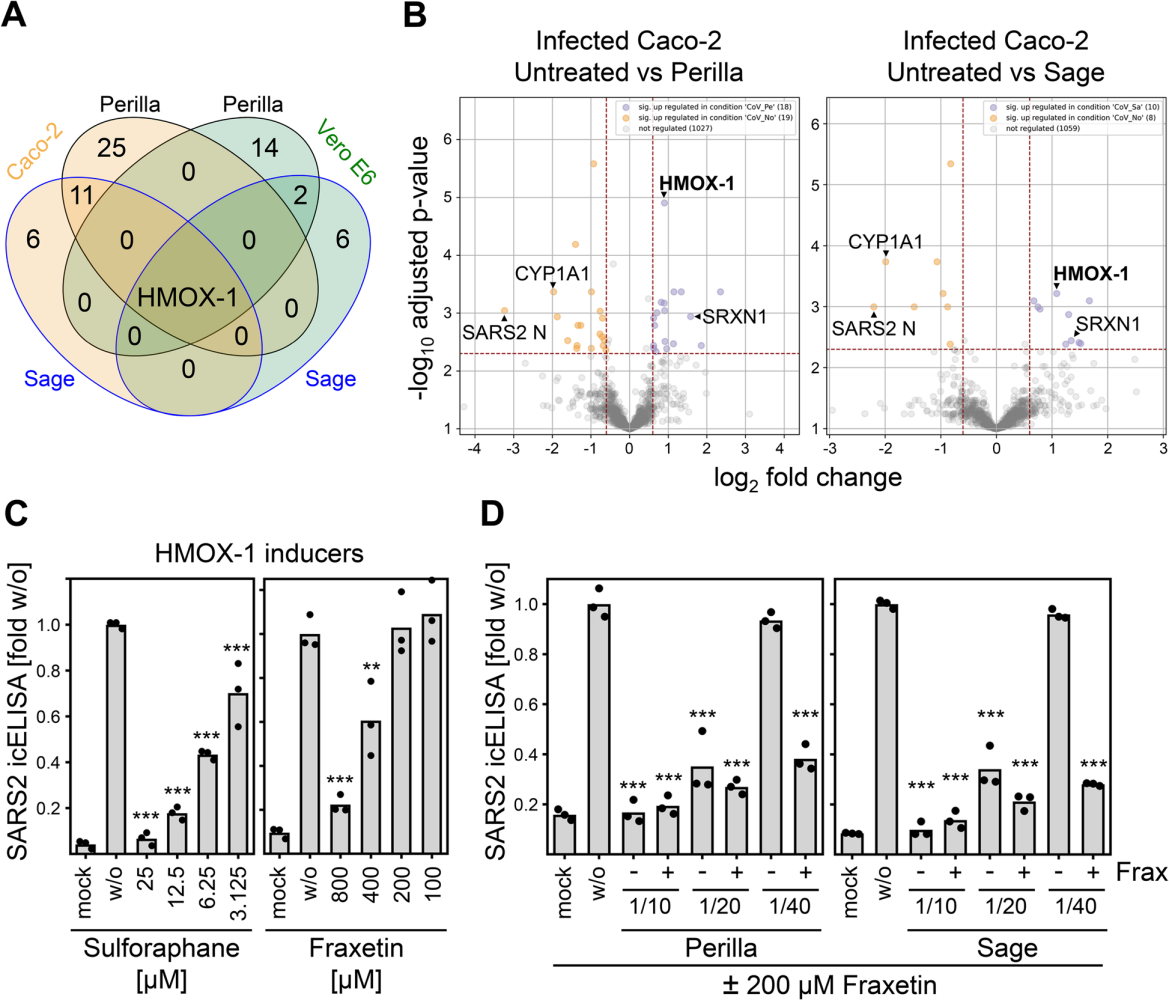
Global MS analyses proposed HMOX-1 induction as anti-SARS-CoV-2 effector mechanism. **A** SARS-CoV-2-infected Vero E6 and Caco-2 cells were treated at 1 h p.i. with perilla or sage infusions for 1 h. At 6 h p.i., cells were lysed and subjected to global MS analysis (see “Methods” section for details). Untreated and infusion-treated cells infected with SARS-CoV-2 were compared (see “Methods” section for details), overlapping significantly regulated proteins are shown. **B** Volcano plots of MS results obtained from infected Caco-2 at 6 h p.i. Normalized data were filtered (at least two unique peptides per protein group required) and proteins with a coefficient of variation < 20.0% over all measurements (also across treatments) were removed (see “Methods” section for details). **C** SARS-CoV-2-infected Vero E6 cells were treated at 1 h p.i. for 1.5 h with the HMOX-1 inducer sulforaphane or fraxetin. Cells were fixed at 24 h p.i. and stained with α-S mAb. Data are expressed as relative change in optical density compared to the untreated control. Each condition was analyzed in triplicate. See Additional file 1 for individual data values. The treated conditions were compared to the untreated control by one-way ANOVA. **, *p* < 0.01. ***, *p* < 0.001. **D** SARS-CoV-2-infected Vero E6 cells were treated at 1 h p.i. for 1.5 h with 200 µM fraxetin and indicated dilutions of perilla or sage infusions. At 24 h p.i., cells were fixed and stained with α-S mAb. Data are expressed as relative change in optical density compared to the untreated control. Each condition was analyzed in triplicate. See Additional file 1 for individual data values. The treated conditions were compared to the untreated control by one-way ANOVA. ***, *p* < 0.001

Next, we sought to determine proteomic changes in cells treated with perilla and sage infusions to advance our understanding of effector mechanisms that lead to the reduction of SARS-CoV-2 replication. To this end, SARS-CoV-2-infected Vero E6 and Caco-2 cells as well as mock controls were treated at 1 h p.i. with perilla and sage infusion for 1 h. At different time points after infection (2, 6, 20 h p.i. in the case of Vero E6 and 2, 6, 30 h p.i. in the case of Caco-2), cells were lysed and subjected to global MS analysis. To be able to detect the mutual interplay between viral and host proteins, we increased the virus amount used for infection to MOI 0.3 and MOI 0.5 for Vero E6 and Caco-2, respectively. This experimental setting might result in overwhelming expression of viral gene products at late times postinfection, but it presumably allows sufficient viral gene expression early after infection to unravel the impact of viral proteins on cellular processes. Overall, we detected and quantified 19 viral proteins and protein forms as well as 3733 cellular proteins in Vero E6 and 4646 cellular proteins in Caco-2 samples by MS. To our knowledge, this is the first reported proteomic dataset investigating effects of herbal extracts such as perilla and sage in cell culture models. To support broad access to the dataset and to allow facile data visualization, we included a browsable ready-to-use Microsoft Excel-based plotting option as supplement (Additional files 12 and 13 for quantified Vero E6 and Caco-2 proteins, respectively). Using these tables, relative abundances of all individual proteins detected by at least two unique peptides can be visualized. Next, we examined the MS data to evaluate whether proteomic changes in host proteins, which have been suggested as host restriction factors (HRFs) and host dependency factors (HDFs) [34–36], could explain the observed antiviral activity. For this purpose, we analyzed all proposed HRFs and HDFs that could be consistently quantified across all investigated conditions. These analyses revealed no significant and consistent upregulation of HRFs and downregulation of HDFs upon treatment with perilla or sage infusions (Additional files 14 and 15: Fig. S11 and S12). Therefore, we searched for proteins significantly regulated at least 1.5-fold upon treatment with perilla or sage infusions in infected cells at 6 h p.i. because this condition most likely includes the relevant changes. For the differential comparisons, the proteins were filtered by the coefficient of variation (CV): all proteins with a CV < 20% over all measurements (also across treatments) were not considered. When comparing the changes induced by treatment with perilla or sage infusions in infected cells at 6 h p.i., heme oxygenase 1 (HMOX-1) was the only protein found to be significantly altered under all 4 conditions (Fig. 5A). HMOX-1 is an enzyme involved in the response to oxidative stress. It catalyzes the oxidative degradation of heme to biliverdin, a precursor of bilirubin, thereby detoxifying free heme [37, 38]. When we examined in detail the individual proteins regulated by perilla and sage infusions, we found additional proteins involved in the oxidative stress response (Fig. 5B and Additional file 16: Fig. S13). As example, Sulfiredoxin 1 (SRXN1), an endogenous antioxidant protein that prevents cell oxidative stress damage, was also upregulated in Caco-2 cells after treatment. Interestingly, levels of CYP1A1 that was recently shown to inhibit HMOX-1-mediated bilirubin formation [39] were decreased in infected Caco-2 cells after treatment with perilla or sage infusions (Fig. 5B and Additional file 16: Fig. S13). This finding confirmed that activity of HMOX-1 was elevated in infected cells treated with perilla or sage infusions. Noteworthy, viral N as well as membrane (M) and ORF7a (7a) proteins were the most strongly downregulated proteins in Caco-2 and Vero E6 cells, respectively, when treated with herbal infusions (Fig. 5B and Additional file 16: Fig. S13), which further corroborates the observed antiviral activity.

To test whether the increase in HMOX-1 protein levels and activity may represent one of the effector mechanisms of herbal infusions to restrict SARS-CoV-2 replication, we examined the antiviral activity of HMOX-1-inducing substances such as sulforaphane and fraxetin. As expected, we observed antiviral activity after short-term treatment with sulforaphane or fraxetin (Fig. 5C). This result raised the question of whether this information could be applied to improve the efficacy of the herbal treatments. HMOX-1 was found to be upregulated about twofold in infected Caco-2 cells (Fig. 5B). Therefore, we tested whether the joint HMOX-1 induction resulting from a combination of suboptimal doses of perilla or sage in conjunction with a dose of fraxetin (200 µM) that was too low to be active against SARS-CoV-2 when applied as a single treatment (Fig. 5C) together elicits antiviral activity in cell culture. Fraxetin and sulforaphane did not show cytotoxic activity—neither alone nor in combination with perilla or sage extracts (data not shown). In fact, combinations of low-dose fraxetin with perilla or sage infusions strongly enhanced the antiviral activity (Fig. 5D).

### Herbal infusions exhibit antiviral activity against SARS-CoV-2 variants of concern

**Fig. 6:**
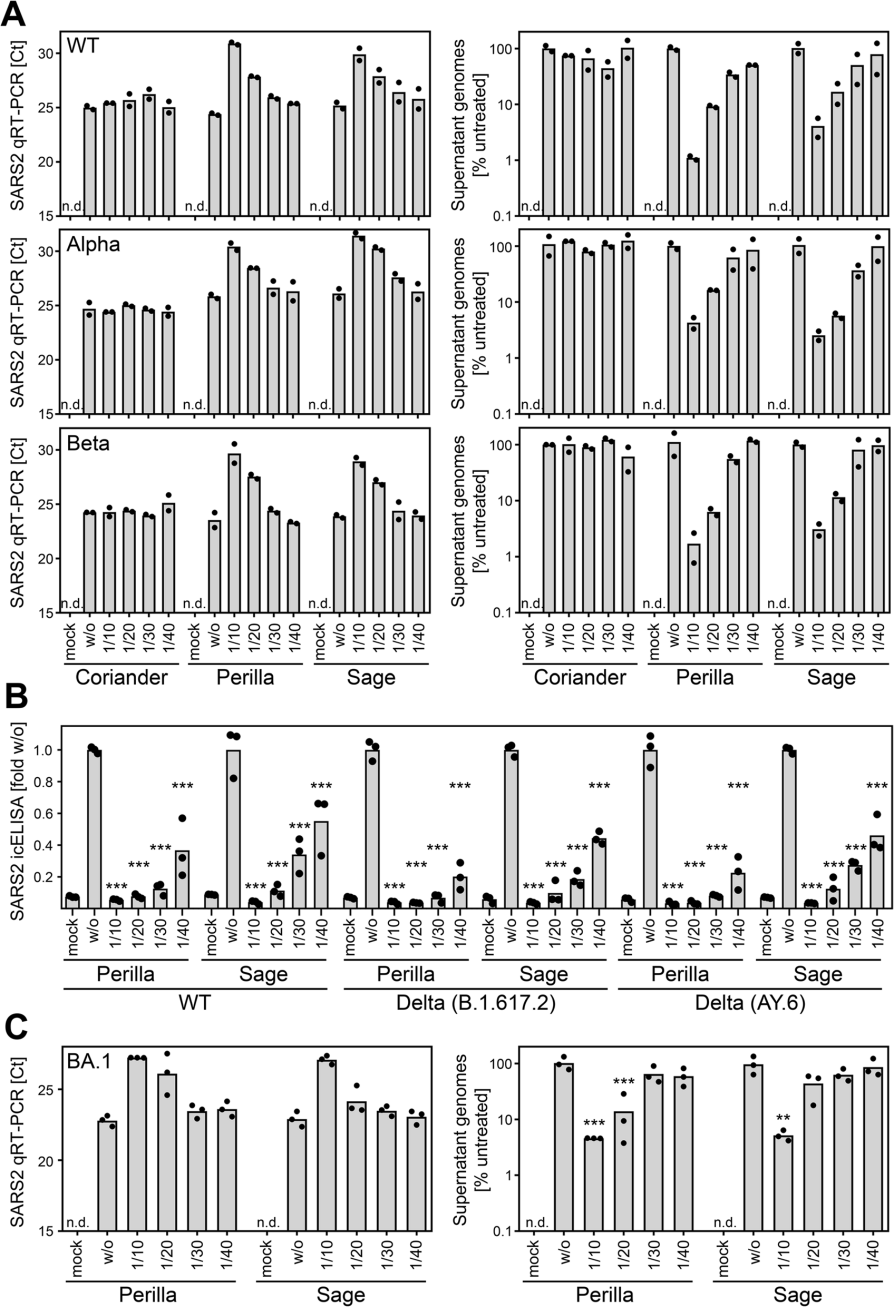
Herbal infusions exhibit antiviral activity against SARS-CoV-2 variants of concern. **A** Vero E6 cells were infected with SARS-CoV-2 wt and the variants of concern Alpha (B.1.1.7) and Beta (B.1.351) after treatment with aqueous infusions of coriander, perilla, or sage at 1 h p.i. for 1 h. SARS-CoV-2 replication was determined by quantification of viral genomes in the supernatant of infected cells at 20 h p.i.. Data are shown in Ct value and calculated relative change in genome copies compared to the untreated control. Each condition was analyzed in duplicate. See Additional file 1 for individual data values. **B** Caco-2 cells were infected with SARS-CoV-2 wt and two distinct isolates of Delta (B.1.617.2 and AY.6) after treatment with aqueous infusions of perilla or sage at 1.5 h p.i. for 1.5 h. SARS-CoV-2 replication was analyzed at 30 h p.i. by icELISA (α-S staining). Data are expressed as relative change compared to the untreated control. Each condition was analyzed in triplicate. See Additional file 1 for individual data values. The treated conditions were compared to the untreated control by one-way ANOVA. ***, *p* < 0.001. **C** Caco-2 cells were infected with SARS-CoV-2 variant of concern Omicron (BA.1) after treatment with aqueous infusions of perilla or sage at 1.5 h p.i. for 1.5 h. SARS-CoV-2 replication was determined by quantification of viral genomes in the supernatant of infected cells at 24 h p.i.. Data are shown in Ct value and calculated relative change in genome copies compared to the untreated control. Each condition was analyzed in triplicate. See Additional file 1 for individual data values. The treated conditions were compared to the untreated control by one-way ANOVA. ***, *p* < 0.001. **, *p* < 0.01

Since the beginning of the pandemic, several SARS-CoV-2 variants emerged. In particular, the variants of concern (VOCs) exhibit strong impact on the course of the pandemic. Therefore, we tested whether the antiviral principle induced by treatment with perilla and sage infusions also counteracts in vitro infections with the VOCs Alpha (B.1.1.7), Beta (B.1.351), Delta (B.1.617.2), and Omicron (BA.1). The E484K mutation present in the S protein of the Beta variant was shown to be an escape mutation impairing the neutralizing capacity of antibodies [40, 41]. This finding was confirmed with our Beta virus isolate. We were not able to stain Beta by icELISA when we used two different monoclonal antibodies both binding to the RBD of S (Additional file 17: Fig. S14A). When using a polyclonal α-RBD antibody, the signal was reduced but not completely lost (Additional file 17: Fig. S14A). In contrast, the recognition by α-N antibodies was not affected (Additional file 17: Fig. S14A). Based on these observations, we used α-N for icELISA detection and quantification of Beta infections. We analyzed the antiviral activity of perilla and sage infusions by icELISA as well as qRT-PCR quantification of viral progeny in the supernatant and observed similar antiviral effects against wild type, Alpha, and Beta (Additional file 17: Fig. S14B, Fig. 6A). We also detected strong antiviral activity of perilla and sage infusions against the Delta variant (Fig. 6B), but not when coriander infusion was used (data not shown). Delta could only be tested in human cells because the P681R mutation in the S protein of Delta almost completely prevents infection of Vero E6 cells (unpublished observation). Similarly, the currently most widespread Omicron variant was assessed in human cells. In our hands, Omicron hardly replicated in Vero E6 cells, and even in human cells, Omicron replicated only to low titers (unpublished observation). For this reason, we used lower input virus doses and did not apply the icELISA readout but used the more sensitive quantification of viral progeny by qRT-PCR. This analysis revealed that perilla and sage infusions also elicited antiviral activity against Omicron (Fig. 6C).

Taken together, aqueous infusions based on perilla and sage exhibit strong in vitro antiviral activity against SARS-CoV-2 including VOCs such as Alpha, Beta, Delta, and Omicron.

## Discussion

The WHO SOLIDARITY trial comprising more than 400 hospitals and 11,000 patients in 30 countries showed that “Remdesivir, Hydroxychloroquine, Lopinavir and Interferon regimens appeared to have little or no effect on hospitalized COVID-19, as indicated by overall mortality, initiation of ventilation and duration of hospital stay” [42]. To a certain extent, this finding may be attributed to the practice of reserving such drugs for critically ill patients for whom they may be applied too late in terms of disease progression. Recently, Merck and Pfizer applied for an emergency use authorization by the US Food and Drug Administration (FDA) for the drugs Molnupiravir and Paxlovid, respectively. Clinical trials showed promising results in reducing the risk of hospitalization or death when given early in the infection, but definitive clinical data are still needed to prove the efficacy of the antiviral treatment candidates. A widely discussed potential solution to the limited efficacy of single therapies is the combination of substances that have a marginal effect on their own. Such “shotgun” treatment regimens have already shown some success [43]. Since herbal teas are ubiquitously available, almost free of charge, and exhibit excellent safety profiles given their consumption as spices, these *Lamiaceae* teas may be applicable as an addition to the very important non-pharmaceutical interventions such as wearing a face cover, proper hygiene, physical distance, and the restriction of social interactions.

Using different SARS-CoV-2 cell culture models, highly permissive Vero E6 cells and human Caco-2 cells, we observed potent antiviral activity of herbal teas produced by boiling leaves of the *Lamiaceae* perilla and sage. A typical cup of tea corresponds to approx. 250 ml of volume and commercial tea bags usually contain 1–3 g of plant material. Our herbal extracts were produced using 15 g of fresh herb leaves boiled in 100 ml of water. Based on a water content of 80–90% of fresh herbs, a 1/20 dilution of these infusions already corresponds to herbal teas prepared from standard tea bags (1–3 g dried tea per 250 ml cup). In human cells, perilla and sage infusions elicited significant antiviral effects at dilutions of 1/20 (0.75 g per liter or ca. 0.1875 g per cup) (Figs. 2 and 3), indicating efficacy at concentrations typically consumed in such herbal teas. Processed perilla leaf extract has also been suggested to elicit virucidal activity on cell-free SARS-CoV-2 virions [44, 45], supporting the reliability of its potential application as universally available herbal tea against COVID-19.

Given that Vero cells are incapable to express type I interferons due to genetic aberrations [28], the antiviral activity observed in Vero E6 cells indicate that perilla and sage extracts elicit their effect independent of interferon induction. The result that the Janus kinase inhibitor ruxolitinib did not revert the antiviral activity elicited by perilla and sage infusions in Caco-2 cells supports this conclusion. Our finding that *Lamiaceae* infusions exhibit antiviral activity in both Vero E6 and Caco-2 cells (Figs. 1 and 2) suggests that S cleavage and the endosomal entry route are at least not the only target of the antiviral mechanism, since SARS-CoV-2 entry into Vero E6 and Caco-2 is mediated by distinct routes [27]. This conclusion is further supported by our observation that sage and perilla teas diminished viral replication, especially when they were applied after infection at a time point at which neutralizing antibodies present in convalescent serum had lost most of their antiviral activity. In such treatment regimens, the herbal teas significantly outperformed NAb-containing serum (Fig. 1E).

Natural products and traditional herbal medicines have often paved the way to the development of novel therapeutic agents. Many drugs were developed based on the structure of natural compounds that exhibit the desired effect. Examples of this approach of drug development include the antimalarial drug artemisinin derived from *Artemisia annua*, the cancer drug taxol derived from *Taxus brevifolia*, and the amoebicide emetine, an isoquinoline alkaloid from *Cephaelis ipecacuanha*. Numerous other drugs were modified from natural compounds such as aspirin and morphine. In fact, half of all drugs approved between 1981 and 2019 were derived from or mimicked a natural compound [46].

Upon selection of candidate compounds based on protein fractionation analyses, we identified caffeic acid, perilla aldehyde, and perillyl alcohol as antiviral substances (Fig. 4). Caffeic acid is a plant-derived compound containing both phenolic and acrylic functional groups. It is abundantly found in natural products and was shown to exhibit antimicrobial and antioxidant activities [47, 48]. The observation that caffeic acid/dihydroxy-cinnamic acid, but not hydroxy-cinnamic acid, acts antiviral (Additional file 10: Fig. S9A) provides insight into the reactive groups and may allow improvement of activity by structural optimization. Perilla aldehyde has gained attention in recent years because of its antioxidant and antifungal properties and its potential to serve as organic fruit and food preservative [49]. Animal studies indicated anticancer and anti-inflammatory activity [50]. Perillyl alcohol has also attracted attention because of its potential anticancer activity [51]. Our data constitute the first experimental evidence for the antiviral activity of these substances against SARS-CoV-2, suggesting that caffeic acid, perilla aldehyde, and perillyl alcohol could be candidates for the development of antiviral agents. Plants of the *Lamiaceae* family have been shown to possess antiviral activity against the retrovirus HIV [52–54]. Similar to our findings with SARS-CoV-2, the anti-HIV activity seems to occur at a post-entry step [55]. It will be very interesting to test the compounds responsible for the anti-SARS-CoV-2 activity of perilla and sage infusions and to elucidate whether the antiviral activity against retroviruses and coronaviruses is mediated by the same or similar mechanisms.

Global MS analyses revealed that both perilla and sage treatments upregulated HMOX-1 abundance and activity. Follow-up experiments showed that upregulation of HMOX-1 is indeed an antiviral effector mechanism inhibiting SARS-CoV-2 infection (Fig. 5). In addition, recently published data show that the HMOX-1 axis is so detrimental for SARS-CoV-2 that it selected for an inhibitory function encoded in the viral Nsp14 protein [56]. This further supports our argument that herein documented *Lamiaceae* herb-induced HMOX-1 induction elicits relevant antiviral activity against SARS-CoV-2. Considering that SARS-CoV-2-induced inflammation [57], coagulopathies [58], and respiratory distress syndrome (ARDS) [59] are major causes of mortality in COVID-19 patients [60, 61], the known protective role of HMOX-1 activation regarding inflammation, inflammation-induced coagulation, lung damage, and ARDS [62–66] might suggest that the herbs and compounds we identified may act in diverse contexts.

We are convinced that our data argue in favor of future clinical studies addressing the question of whether herbal teas based on perilla, mint, thyme, or sage may either be able to prophylactically reduce infections and/or offer therapeutic benefits when administered concomitantly with the standard treatment. In such clinical studies, potential side effects of herbal tea consumption such as allergic reactions [67] and causing of toxicity to the fetus during pregnancy (https://www.cdc.gov/pregnancy/meds/treatingfortwo/facts.html) must be considered. Obviously, the consumption of herbal teas cannot and should not replace non-pharmaceutical interventions or clinically approved drugs.

## Conclusions

Herbal teas based on perilla and sage exhibit antiviral activity against SARS-CoV-2 including variants of concern such as Alpha, Beta, Delta, and Omicron. Using global MS analyses, we identified HMOX-1 as potential therapeutic target. Given that perilla and sage have been suggested as treatment options for various diseases, our dataset may constitute a valuable resource also for future research beyond virology. In addition, our study identified caffeic acid, perilla aldehyde, and perillyl alcohol as potential antiviral drugs that deserve further investigations.

## Methods

### Materials and correspondence

Further information and requests for resources and reagents should be directed to and will be fulfilled by Mirko Trilling (Mirko.Trilling@uk-essen.de).

### Cells, viruses, and infection

Vero E6 (ATCC CRL-1586) and Caco-2 (ATCC HTB-37) were grown in high-glucose Dulbecco’s minimal essential medium (DMEM [Gibco 41,966–029]) and Roswell Park Memorial Institute 1640 (RPMI-1640 [Gibco 21,875–034]), respectively, supplemented with 10% (v/v) FCS, penicillin, and streptomycin. Calu-3 (ATCC HTB-55) were cultivated in minimal essential medium (MEM [Gibco 31,095–029]) supplemented with 10% (v/v) FCS, 1 mM sodium pyruvate, penicillin, and streptomycin. All cells were kept at 37 °C in an atmosphere of 5% CO_2_. All cells were tested for mycoplasma contamination and only used when free of mycoplasma.

The SARS-CoV-2 strains B.1, B1.1.232, Alpha (B.1.1.7), Beta (B.1.351), Delta (B.1.617.2 and AY.6), and Omicron (BA.1) were isolated from patient samples obtained in May 2020, March to May 2021, and December 2021. For the isolation, permissive cells were incubated with virus-containing clinical nasopharyngeal swab samples until CPE was observed. All strains except Delta and Omicron were amplified in Vero E6 cells. The Delta isolates B.1.617.2 and AY.6 as well as the Omicron isolate BA.1 were isolated and amplified using Calu-3 cells. All SARS-CoV-2 isolates were analyzed by Next Generation Sequencing (data not shown) and classified with the help of GISAID and assigned into clades according to pangolin [68–71]. The authors made use of the outbreak.info [72]. Viral titers were determined by 50% tissue culture infectious dose (TCID50) titration. The virus isolation has been approved by the ethics committee of the medical faculty of the University of Duisburg-Essen (20–9511-BO and 20–9512-BO). HSV-1-ΔgE-GFP was generated and described [73] by the laboratory of Prof. David C Johnson (Oregon Health & Science University, USA). With Prof. Johnson’s written permission, we received the virus from Prof. Hartmut Hengel (University of Freiburg, Germany).

### Generation of aqueous infusions of herbs

The herbal infusions were prepared by boiling up 15 g of fresh herbal leaves in 100 ml of water and subsequent simmering at 60 °C for 2 h. The infusions were stored overnight at 4 °C before the leaves were removed and the aqueous solutions were sterile-filtered (200 µM filter, Whatman/GE Healthcare). Afterwards, the herbal infusions were stored in aliquots at − 80 °C. For the infusions based on dried herbs, 3 g of material per 100 ml was utilized. The 10-min infusion was prepared by boiling up dried sage leaves in water (30 g per liter) and subsequent incubation for 10 min before the herb was removed. Sterile-filtered aliquots were then stored at − 80 °C. Infusions of frozen and re-thawed herbal material were prepared in the same manner as those based on fresh herbs. The concentration of 150 g of herbal material per liter was calculated based on the fresh weight before freezing. The sources of supply for the herbal leaves and plants are as follows: coriander, sage and mint, farmer’s market (Essen, Germany); red and green perilla plants, online vendor Naturkraeutergarten (Kleinich, Germany); bi-color perilla plant, home-grown; dried red perilla, home-dried; dried green perilla, Keiko Shiso Finest Selection (Japan); dried sage and dried thyme, vom-Achterhof Bio-Salbei/Bio-Thymian (Uplengen, Germany), mint tea bags, Meßmer Pfefferminze (Germany).

### In-cell-ELISA (icELISA)

For the quantification of viral protein amounts in infected cells, an icELISA was applied. A detailed icELISA protocol is provided in [26]. Briefly, cells were infected with SARS-CoV-2 and fixed after 20 or 30 h of infection with 4% (w/v) paraformaldehyde/PBS. Cells were permeabilized with 1% (v/v) Triton-X-100/PBS and blocked with 3% (v/v) FCS/PBS. The primary antibody was added and incubated for 2 h at room temperature (RT) or overnight at 4 °C. Peroxidase-labelled secondary antibody was incubated for 1–2 h. Washing steps were performed with 0.05% (v/v) Tween-20/PBS. Tetramethylbenzidin (TMB) substrate was added to visualize the enzyme reaction. The reaction was stopped with 0.5 M HCl before the absorbance was determined using a microplate multireader and MicroWin software (Mithras2 LB 943; Berthold Technologies). The resulting data were analyzed using Excel and GraphPad Prism software. The α-S mAb (kindly provided by Peter Miethe, fzmb, Bad Langensalza, Germany), α-N mAb (ABIN6952435), and POD-coupled secondary antibodies (Dianova) were used.

### Dose–response curves of antiviral activity

To enable comparison among different icELISA measurements and experiments, we included on every plate a virus calibration curve. Residual infectivity after treatment was calculated using the formula computed from the calibration curve (see Additional file 2: Fig. S1 as an example). Dose–response curves were compiled based on the relative change in infectivity compared to the untreated control.

### Immunofluorescence microscopy

Cells were infected with SARS-CoV-2 and fixed after 20 or 30 h of infection using 4% (w/v) paraformaldehyde/PBS for > 2 h before they were discharged from the BSL-3 laboratory. Cells were permeabilized with 1% (v/v) Triton-X-100/PBS and blocked with 3% (v/v) FCS/PBS. SARS-CoV-2 infection was visualized by use of α-S mAb (kindly provided by Peter Miethe, fzmb, Bad Langensalza, Germany) and Cy2-conjugated goat anti-mouse IgG (Dianova). Nuclei were counterstained with 4′6-diamidino-2-phenylindole (DAPI; Sigma). Fluorescence was visualized using a THUNDER Imager 3D Cell Culture (Leica). Image analysis and processing were performed with LAS X Premium imaging software (Leica).

### Quantitative reverse-transcription PCR (qRT-PCR)

SARS-CoV-2 progeny was analyzed by quantification of viral RNA extracted from the culture supernatants of infected cells using the INSTANT Virus RNA/DNA Kit (Analytik Jena, Germany). Viral RNA was quantified by diagnostic qRT-PCR targeting the SARS-CoV-2 genes S and E (RealStar ® SARS-CoV-2 RT-PCR kit, Altona, Hamburg, Germany). In the case of Omicron, undiluted RNA preparations were used as template for qRT-PCR, for all other variants, a 1:50 dilution was used.

### Interferons, inhibitors, substances, and size exclusion

Human IFNα2 was purchased from PBL Assay Science (#11101) and IFNβ from Peprotech (#300-02BC). Remdesivir and ruxolitinib were obtained from Cayman Chemicals (#30354) and Cell Guidance Systems (#SM87-10), respectively. The substances cinnamic acid, hydroxy-cinnamic acid, and dihydroxy-cinammic acid/caffeic acid were purchased from Sigma (#8002350250, #8002370050, #8220290010). Perilla aldehyde and perillyl alcohol were also obtained from Sigma (#W355704 and #218391). Size exclusion and protein fractionation of components of herbal infusions were conducted by use of Amicon 100 K, 30 K, 10 K, and 3 K filters (Sigma #UFC510024, #UFC501096, #UFC503096, #Z740183-96EA). The fraction of proteins > 1 kDa was obtained by dialysis using the Mini Dialysis Kit 1 kDa (Sigma #GE80-6483–94). All kits were applied according to the manufacturer’s instructions.

### Sample preparation for MS

For protein extract preparation, 80–90% confluent cell monolayers of a T25 flask of Vero E6 or Caco-2 cells were harvested using a cell scraper. Medium and cells were transferred to a 15-ml tube and centrifuged for 3 min at 350 g. Pellets were washed twice with 10 ml PBS, resuspended in 400 µl of lysis buffer (50 mM Tris, 150 mM NaCl, 1% [w/v] SDS, pH 7.8 supplemented with one cOmplete mini EDTA-free protease inhibitor tablet as well as one PhosSTOP tablet [both Roche] per 10 ml volume of lysis buffer), and transferred into 1.5-ml tubes. Lysates were incubated for 30 min at 4 °C before they were subjected to virus inactivation at 70 °C for 10 min followed by 10 min at 95 °C. After ultrasonic treatment for 1 min, cell lysates were stored at − 80 °C until further analysis. Six microliters benzonase (27 units/µl, Merck) and 1 µl MgCl_2_ (2.5 mM final concentration) were added per sample, followed by incubation at 37 °C for 30 min. Protein concentration was determined by a bicinchoninic acid assay (Pierce), and 100 µg protein lysate per replicate (*n* = 5 for all analyzed conditions) was processed further as described below.

### Positive pressure filter-aided sample preparation (FASP) in 96-well format

Cysteines were reduced using 10 mM dithiothreitol (Roche) at 56 °C for 30 min and alkylated in the presence of 25 mM iodoacetamide (Sigma) for 30 min at RT in the dark. Samples were diluted at least 1:4 in 8 M urea (Sigma-Aldrich) dissolved in 100 mM Tris–HCl, pH 8.5 (Applichem), and transferred to a 30-kDa AcroPrep Omega filter membrane plate (PALL, New York, USA, purchased via VWR, Hannover, Germany, REF 8035/518–0028) in a blocked randomized order, which was also kept for subsequent LC–MS measurements. The filter plate was placed on top of a 2.2-ml MegaBlock collection plate (Sarstedt, Nümbrecht, Germany) and the liquid of the protein solution was forced through the filter using a Resolvex A200 (Tecan, Männedorf, Switzerland) connected to nitrogen gas (N_2_, 5.5 bar, purity 4.8 or higher, Linde, Düsseldorf, Germany) using a relative pressure of 20% of the low profile setting. Subsequently, the dispensing function of the A200 was used to wash the filter twice with 200 µl 8 M urea buffer in Tris–HCl pH 8.5 and twice with 200 µl 50 mM ammonium bicarbonate (ABC, Fluka). After each washing step, the liquid was forced through the filter using the same pressure profile as for loading. Afterwards, the plate was centrifuged for 2 min at RT and 1000 g to remove residual drops under the membrane. For digestion, 100 µl digestion buffer were added comprising 100 mM urea, 50 mM ABC, and 2 mM CaCl_2_ (Merck) including sequencing-grade trypsin (Promega, sequencing-grade modified trypsin) in a concentration to meet a 1:3 (w/w) enzyme-to-sample ratio. After incubation for 16 h at 37 °C, the digested protein fraction was forced through the filter and collected in a 500-µl LoBind plate (Eppendorf, Hamburg, Germany). The filter was further washed with 100 µl 50 mM ABC followed by 100 µl H_2_O, both wash steps were also collected in the LoBind plate. Then, tryptic digestion was stopped by reducing the pH < 2 through the addition of trifluoroacetic acid (TFA; Biosolve, Valkenswaard, Netherlands) to a final concentration of 1% (v/v). Aliquots were transferred to 700 µl glass-vial plates (Waters, Eschborn, Germany) for injection on a monolithic column HPLC (for tryptic digestion quality control) [74]. Based on the results, 4–5 replicates per condition were further analyzed by LC–MS.

### LC–MS in data-dependent acquisition-mode (DDA)

LC–MS was conducted using an UltiMate 3000 RSLCnano ProFlow UPLC system operated by Chromeleon Client 6.80 and online-coupled to a Q Exactive HF MS operated by Tune application 2.8 SP1 and Thermo Scientific Xcalibur 3.0.63 (both Thermo Scientific, Dreieich, Germany, including HPLC columns). Employed solvents were LC–MS grade or higher (Biosolve, Valkenswaard, Netherlands). In total, 825 ng of tryptic peptides per LC–MS injection was analyzed. Samples were loaded on a trapping column (Acclaim PepMap C18, 0.1 × 20 mm, 5 µm, 100 Å) for 3 min in 0.1% TFA at a flow rate of 30 µl/min. Then, the trapping column was switched in line with the analytical column (Acclaim PepMap C18; 0.075 × 500 mm, 2 µm, 100 Å) and peptides were separated at a flow rate of 250 nl/min using a 102-min linear gradient of buffer B (84% v/v acetonitrile [ACN], 0.1% v/v formic acid [FA]) in buffer A (0.1% v/v FA) ranging from 3 to 25% B, followed by a 10-min linear gradient ranging from 25 to 35% B, washing steps and reconditioning of the analytical column to 3% B. Both columns were kept at 60 °C temperature.

The UPLC system was coupled to the Q Exactive HF MS via a NSI Source (Thermo). Coated emitters (Silica Tip, 20 μm inner diameter, 10 μm tip inner diameter, New Objectives, Woburn, MA) and a static voltage of 1.8 kV were applied for electrospray ionization, and ion transfer tube temperature was set to 250 °C. The MS was operated in data-dependent acquisition (DDA) mode at positive polarity; all spectra were acquired in profile mode with survey scans acquired at a resolution of 60,000 followed by 15 MS/MS scans at a resolution of 15,000 (top15). Precursor ions were selected for MS/MS by intensity, isolated in a 1.6 m/z window and subjected to fragmentation by higher-energy collision-induced dissociation using a normalized collision energy (NCE) of 27. Automatic gain control target values were set to 10^6^ and 5 × 10^4^ and the maximum ion injection was set to 120 ms and 50 ms for MS and MS/MS, respectively. Precursor masses were excluded from re-fragmentation for 20 s (dynamic exclusion).

### Protein identification and relative quantification with Proteome Discoverer

DDA files were processed with Proteome Discoverer 2.4 (Thermo Scientific) using Spectrum Files RC and Sequest HT nodes as database search algorithm and Percolator [75] in conjunction with Peptide validator and Protein FDR validator nodes for adjusting the false discovery rate to 1% on PSM, peptide, and protein levels. Database search was conducted against Uniprot homo sapiens database for Caco-2 (UP000005640, 20,376 entries, retrieved in November 2019) or Chlorocebus sabaeus database for Vero E6 (UP000029965, 19,230 entries, retrieved in January 2021) in conjunction with the SARS-CoV-2 database (UP000464024, 14 entries, retrieved in January 2021) supplemented with reported putative novel ORFs [76], common contaminants, and an additional fasta file containing the amino acid sequence of indexed retention time peptides added as an internal standard [77] by the Sequest HT search engine with the following parameters: error tolerances of 10 ppm and 0.02 Da for precursor and fragment, trypsin (full) as enzyme with a maximum of two missed cleavage sites, oxidation of Met as variable modification (+ 15.995 Da) and carbamidomethylation of Cys (+ 57.021 Da) as fixed modification. Full settings are provided as additional material via ProteomeXchange. Quantification was performed using the Minora feature detector node in conjunction with the Feature mapper node with a maximum retention time shift of 10 min and mass tolerance of 10 ppm for chromatographic alignment and minimum signal to noise threshold of 10 for feature linking and mapping. Precursor Ions Quantifier node accepted only unique peptides for quantification while considering protein groups for peptide uniqueness with disabled scaling and normalization to total peptide amount. Sample abundances of the connected peptide groups were summed to calculate protein abundances. Common contaminant proteins and proteins with less than two unique peptides per protein group were filtered out. Normalized protein abundances were used for further data analysis.

### Analysis of MS data

For all analyses, only proteins with a maximum of one missing replicate value per respective condition were considered. Lightweight normalizing and testing tool (LNTT) [78] was used for the creation of volcano plots in Fig. 5 and Additional file 11: Fig. S10. Proteome Discoverer normalized (total protein normalization) data were filtered (at least two unique peptides per protein group required) and proteins with a coefficient of variation < 20.0% over all measurements (also across treatments) were removed. Independent two-sided two-sample Welch’s *t*-test was performed for remaining proteins. Computed *p* values were adjusted using the Benjamini and Hochberg multiple test correction (https://www.jstor.org/stable/2346101) and results were plotted by LNTT. Gene names for labeling were retrieved by accession numbers using Uniprot API. For heatmap analysis, only proteins quantified across all conditions and time points were considered for Vero E6 and Caco-2 samples. Displayed order of the proteins was sorted accordingly to the mean value for the respective 6 h p.i.

### Statistical analysis

Statistical significance was determined using one-way ANOVA as described in the figure legends. A *p* value of < 0.05 was considered statistically significant. *, *p* value < 0.05. **, *p* value < 0.01. ***, *p* value < 0.001. Calculation of *p* values for analysis of MS data are described above. IC50 values were calculated using GraphPad Prism by nonlinear regression.

## Supplementary Information

**Additional file 1.** Individual data values. Excel table displaying individual data values of graphs with *n* <6 data points per condition.

**Additional file 2: Fig. S1.** Representative example of the virus calibration curves (included on every plate) applied to calculate the residual infectivity after treatment. **A** icELISA data (α-S staining) of SARS-CoV-2-infected Vero E6 cells after treatment with herbal infusions. Each condition was analyzed in triplicate. See Additional file 1 for individual data values. **B** icELISA data of the virus calibration curve. Each condition was analyzed in duplicate. See Additional file 1 for individual data values. **C** The formula of the calibration curve from the same plate was applied to calculate the residual PFU after treatment.

**Additional file 3: Fig. S2.** Two different clinical SARS-CoV-2 isolates exhibit almost identical susceptibilities towards perilla and sage. **A, B** Pooled icELISA data of 3 (**A**, SARS-CoV-2 variant B.1) and 5 (**B**, SARS-CoV-2 variant B.1.1.232) independent experiments of SARS-CoV-2-infected Vero E6 cells after treatment with herbal infusions and icELISA using α-S or α-N mAbs for staining. Data are expressed as relative change in optical density compared to the untreated control. The perilla- and sage-treated conditions were compared to the corresponding coriander-treated condition (same dilution) by one-way ANOVA. **, *p*<0.01. ***, *p*<0.001.

**Additional file 4: Fig. S3.** Preserved sage and perilla leaves retain bioactive compounds. **A** The one-hour treatment with the herbal infusions is not cytotoxic. Vero E6 cells were treated with indicated dilutions of herbal infusions in parallel to an infection experiment. At 18 h post treatment, cell viability was analyzed by Orangu cell counting solution (Cell Guidance Systems). See Additional file 1 for individual data values. **B** Representative dose-response curves of SARS-CoV-2-infected Vero E6 cells (2000 PFU per well) after treatment with herbal infusions. Upper panel depicts the results for 1 h of treatment, lower panel the results for 30 min of treatment. Data are expressed as relative change in infectivity compared to the untreated control. Each condition was analyzed in triplicate. The perilla- and sage-treated conditions were compared to the corresponding coriander-treated condition (same dilution) by one-way ANOVA. **, *p*<0.01. ***, *p*<0.001. **C** Representative dose-response curves of SARS-CoV-2-infected Vero E6 cells after treatment with aqueous infusions generated from fresh or dried red perilla, green perilla, or sage for 1 h. Each condition was analyzed in triplicate. All conditions were compared to the untreated control by one-way ANOVA. *, *p*<0.05. **, *p*<0.01. ***, *p*<0.001. **D** Representative dose-response curves of SARS-CoV-2-infected Vero E6 cells after treatment with aliquots of herbal infusions that were thawed once or twice. Each condition was analyzed in triplicate. All conditions were compared to the untreated control by one-way ANOVA. *, *p*<0.05. ***, *p*<0.001. **E** Representative dose-response curves of SARS-CoV-2-infected Vero E6 cells after treatment with aqueous infusions generated from fresh or frozen red or green perilla. Each condition was analyzed in triplicate. All conditions were compared to the untreated control by one-way ANOVA. ***, *p*<0.001. **F** Representative dose-response curves of SARS-CoV-2-infected Vero E6 cells after treatment with herbal infusions generated by overnight or 10-min extraction of dried sage (see “Methods” section for details). Each condition was analyzed in triplicate. All conditions were compared to the untreated control by one-way ANOVA. ***, *p*<0.001. See Additional file 1 for individual data values. IC50 values were calculated by nonlinear regression using GraphPad Prism. The 1/10 dilutions of perilla and sage extracts contained 351 (red) to 412 (bi-color), and 211 ng, respectively, caffeic acid in 10 µl.

**Additional file 5: Fig. S4.** Perilla and sage elicit* in vitro* antiviral activity when administered prior to infection. **A** Scheme of the experimental setup for the *in vitro* analysis of prophylactic effects against SARS-CoV-2. **B** Pooled icELISA data of 6 independent experiments of SARS-CoV-2-infected Vero E6 cells after pre-treatment with herbal infusions using two distinct SARS-CoV-2 isolates (B.1 and B.1.1.232) for infection of Vero E6 and α-S or α-N mAbs for staining. Data are expressed as relative change in optical density compared to the untreated control. The perilla- and sage-treated conditions were compared to the corresponding coriander-treated condition (same dilution) by one-way ANOVA. *, *p*<0.05. **, *p*<0.01. ***, *p*<0.001. **C, D** Comparison of pre-treatment and treatment (-1 to 0 h p.i. versus 1 to 2 h p.i.) of SARS-CoV-2-infected Vero E6 cells (2000 PFU per well). Each condition was analyzed in triplicate. See Additional file 1 for individual data values. All conditions were compared to the untreated control by one-way ANOVA. **, *p*<0.01. ***, *p*<0.001. **E** Vero E6 cells were infected with SARS-CoV-2 (2000 PFU per well). At 1 or 4 h p.i., cells were treated with perilla infusion for 1.5 h. At 20 h p.i., supernatant was collected for RNA preparation and subsequent qRT-PCR analysis. qRT-PCR data are shown in Ct value and calculated relative change in genome copies compared to the untreated control. Each condition was analyzed in duplicate. See Additional file 1 for individual data values. The perilla-treated conditions were compared to the untreated control by one-way ANOVA. *, *p*<0.05. **, *p*<0.01. ***, *p*<0.001.

**Additional file 6: Fig. S5.** Perilla and sage confer protection against SARS-CoV-2 infection in human cells. **A** Scheme of the applied experimental setup. **B** icELISA data (α-S staining) of SARS-CoV-2-infected Caco-2 cells (2000 PFU per well). Data are expressed as relative change in optical density compared to the untreated control. Each condition was analyzed in triplicate. See Additional file 1 for individual data values.

**Additional file 7: Fig. S6.** Longer treatment periods enhances remdesivir-mediated protection against SARS-CoV-2 infection. **A, B** Pooled icELISA data of 2 independent experiments of SARS-CoV-2-infected Vero E6 (**A**) and Caco-2 (**B**) cells after treatment with remdesivir at 1 h p.i for 1 h p.i. or for the complete time period before fixation. Cells were fixed at 20 or 24 h p.i. and stained with α-S (Vero E6) or α-N mAb (Caco-2). Each condition was analyzed in triplicate. The treated conditions were compared to the untreated control by one-way ANOVA. *, *p*<0.05. **, *p*<0.01. ***, *p*<0.001.

**Additional file 8: Fig. S7.** Size exclusion and protein fractionation analyses of perilla infusion disclosed compounds <10 kDa as active components. Pooled icELISA data (α-S staining) of 2 independent experiments of SARS-CoV-2-infected Vero E6 cells after treatment with indicated fractions of perilla infusion. Data are expressed as relative change in optical density compared to the untreated control. Protein fractionation of perilla infusion components was conducted by use of Amicon 100K, 30K, and 10K filters. Each condition was analyzed in triplicate. See Additional file 1 for individual data values. Dilutions and concentrations by the fractionation steps were considered. All conditions were compared to the untreated control by one-way ANOVA. ***, *p*<0.001. **A** Perilla flow-through of Amicon 100K, 30K, and 10K filters were analyzed. **B** Perilla fractions >100 kDa, >30 kDa, and >10 kDa were analyzed.

**Additional file 9: Fig. S8.** Size exclusion and protein fractionation analyses of perilla and sage infusions disclosed compounds <3 kDa as active components. **A, B** icELISA data of SARS-CoV-2-infected Vero E6 cells after treatment with indicated fractions of herbal infusions (**A**, perilla; **B**, sage) at 1 h p.i. for 1 h. Cells were fixed at 20 h p.i. and stained with α-S mAb. Data are expressed as relative change in optical density compared to the untreated control. Each condition was analyzed in triplicate. See Additional file 1 for individual data values. Protein fractionation of herbal infusion components was conducted by use of Amicon 10K and 3K filters. The fraction of proteins >1 kDa was obtained by dialysis. Dilutions and concentrations by the fractionation steps were considered. The treated conditions were compared to the untreated control by one-way ANOVA. **, *p*<0.01. ***, *p*<0.001.

**Additional file 10: Fig. S9.** Dihydroxy-cinnamic acid, perilla aldehyde, and perillyl alcohol are antiviral components of herbal infusions. **A, B** icELISA data of SARS-CoV-2-infected Caco-2 cells after treatment with indicated substances at 1.5 h p.i. for 1.5 h. Data are expressed as relative change in optical density compared to the untreated control. Each condition was analyzed in triplicate. All treated conditions were compared to the untreated control by one-way ANOVA. *, *p*<0.05. ***, *p*<0.001.

**Additional file 11: Fig. S10.** Treatment with mint infusions elicit antiviral activity *in vitro*. **A **Comparison of pre-treatment and treatment (-1 to 0 h p.i. versus 1 to 2 h p.i.) of SARS-CoV-2-infected Vero E6 cells (2000 PFU per well). Each condition was analyzed in triplicate. See Additional file 1 for individual data values. All conditions were compared to the untreated control by one-way ANOVA. *, *p*<0.05. **, *p*<0.01. ***, *p*<0.001. **B** Representative dose-response curves of SARS-CoV-2-infected Vero E6 cells after treatment with aliquots of herbal infusions that were thawed once or twice. Each condition was analyzed in triplicate. See Additional file 1 for individual data values. All conditions were compared to the untreated control by one-way ANOVA. *, *p*<0.05. ***, *p*<0.001.

**Additional file 12.** Microsoft Excel-based plotting option for relative abundances of all individual proteins detected by MS in Vero E6 samples by at least two unique peptides.

**Additional file 13.** Microsoft Excel-based plotting option for relative abundances of all individual proteins detected by MS in Caco-2 samples by at least two unique peptides.

**Additional file 14: Fig. S11.** Herbal infusion-induced changes of proposed host restriction and host dependency factors in Vero E6 cells. SARS-CoV-2-infected Vero E6 cells as well as mock controls were treated at 1 h p.i. with perilla and sage infusion for 1 h. At 2, 6, and 20 h p.i., cells were lysed and subjected to global MS analysis. Each condition was analyzed in quintuplicate. Proposed HRFs and HDFs [34, 35, 36],which were consistently quantified across all investigated conditions, were analyzed regarding the changes induced by treatment with perilla or sage infusions. The ratio of the treated condition to the respective untreated condition is shown as log_2_fold change.

**Additional file 15: Fig. S12.** Herbal infusion-induced changes of proposed host restriction and host dependency factors in Caco-2 cells. SARS-CoV-2-infected Caco-2 cells as well as mock controls were treated at 1 h p.i. with perilla and sage infusion for 1 h. At 2, 6, and 30 h p.i., cells were lysed and subjected to global MS analysis. Each condition was analyzed in quadruplicate or quintuplicate. Proposed HRFs and HDFs [34, 35, 36],which were consistently quantified across all investigated conditions, were analyzed regarding the changes induced by treatment with perilla or sage infusions. The ratio of the treated condition to the respective untreated condition is shown as log_2_fold change.

**Additional file 16: Fig. S13.** Perilla and sage infusion-induced changes at 6 h p.i. in infected Caco-2 and Vero E6 cells. **A-D** Volcano plots of MS results obtained from infected Caco-2 (**A, B**) or Vero E6 (**C, D**) at 6 h p.i. Proteome Discoverer normalized data were filtered (at least two unique peptides per protein group required) and proteins with a coefficient of variation <20.0% over all measurements (also across treatments) were removed (see “Methods” section for details).

**Additional file 17: Fig. S14.** Herbal infusions exhibit antiviral activity against SARS-CoV-2 variants of concern. **A** Vero E6 cells were infected with graded doses of SARS-CoV-2 wt and Beta (B.1.351). At 20 h p.i., cells were fixed and analyzed by icELISA using different antibodies recognizing the receptor-binding domain of Spike (RBD) or the nucleocapsid protein (N). See Additional file 1 for individual data values. **B** Representative dose-response curves of Vero E6 cells infected with SARS-CoV-2 wt and variants of concern (Alpha/B.1.1.7 and Beta/B.1.351) after treatment with aqueous infusions of coriander, perilla, or sage at 1 h p.i. for 1 h. SARS-CoV-2 replication was analyzed at 20 h p.i. by icELISA (α-N staining). Data are expressed as relative change in infectivity compared to the untreated control. Each condition was analyzed in triplicate. See Additional file 1 for individual data values. The comparison of the herb-treated samples of SARS-CoV-2 to the untreated control by one-way ANOVA showed for all dilutions of coriander no significance. The perilla- and sage-treated conditions of SARS-CoV-2 were also compared to the corresponding coriander-treated condition (same dilution) and these results are depicted in the diagram. *, *p*<0.05. **, *p*<0.01. ***, *p*<0.001.

## Data Availability

All data generated or analyzed during this study are included in this published article and its additional files. The MS proteomics data have been deposited to the ProteomeXchange Consortium [79] via the PRIDE [80] partner repository with the dataset identifier PXD030742.

## Abbreviations

ARDS: Respiratory distress syndrome
CV: Coefficient of variation
COVID-19: Coronavirus disease 2019
FDA: US Food and Drug Administration
(H)CoV: (Human) coronavirus
HDF: Host dependency factor
HIV: Human immunodeficiency virus
HMOX-1: Heme oxygenase 1
h p.i.: Hours post infection
HRF: Host restriction factor
icELISA: In-cell-ELISA
IFN: Interferon
MOI: Multiplicity of infection
MS: Mass spectrometry
N: Nucleocapsid protein
NAbs: Neutralizing antibodies
PFU: Plaque-forming unit
SARS-CoV-2: Severe acute respiratory syndrome coronavirus 2
S: Spike
VOC: Variant of concern
