# Supplementary figures and images for: Identification of herbal teas and their compounds eliciting antiviral activity against SARS-CoV-2 in vitro
Source: BMC Biol. 2022 Nov 30;20:264. doi: 10.1186/s12915-022-01468-z (PMC9708519; doi:10.1186/s12915-022-01468-z)

Additional file 2: Figure S1

A

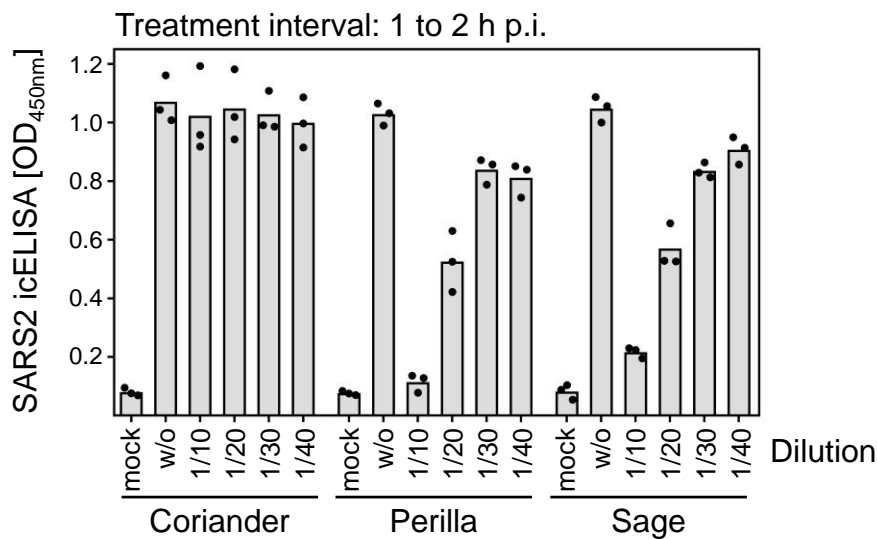

B

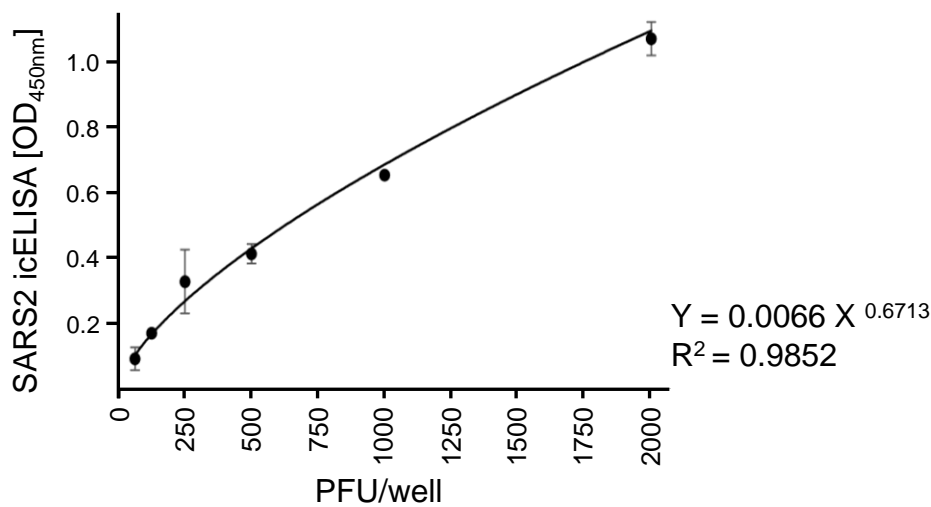

C

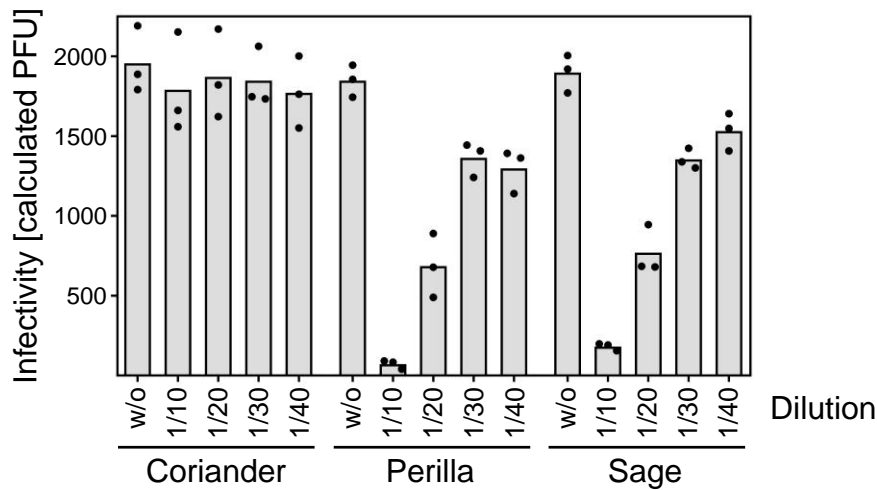

Supplement: Supplementary file 2 — Additional file 2: Fig. S1. Representative example of the virus calibration curves (included on every plate) applied to calculate the residual infectivity after treatment. A icELISA data (α-S staining) of SARS-CoV-2-infected Vero E6 cells after treatment with herbal infusions. Each condition was analyzed in triplicate. See Additional file 1 for individual data values. B icELISA data of the virus calibration curve. Each condition was analyzed in duplicate. See Additional file 1 for individual data values. C The formula of the calibration curve from the same plate was applied to calculate the residual PFU after treatment. [file 12915_2022_1468_MOESM2_ESM.pdf]

Additional file 3: Figure S2

**A**

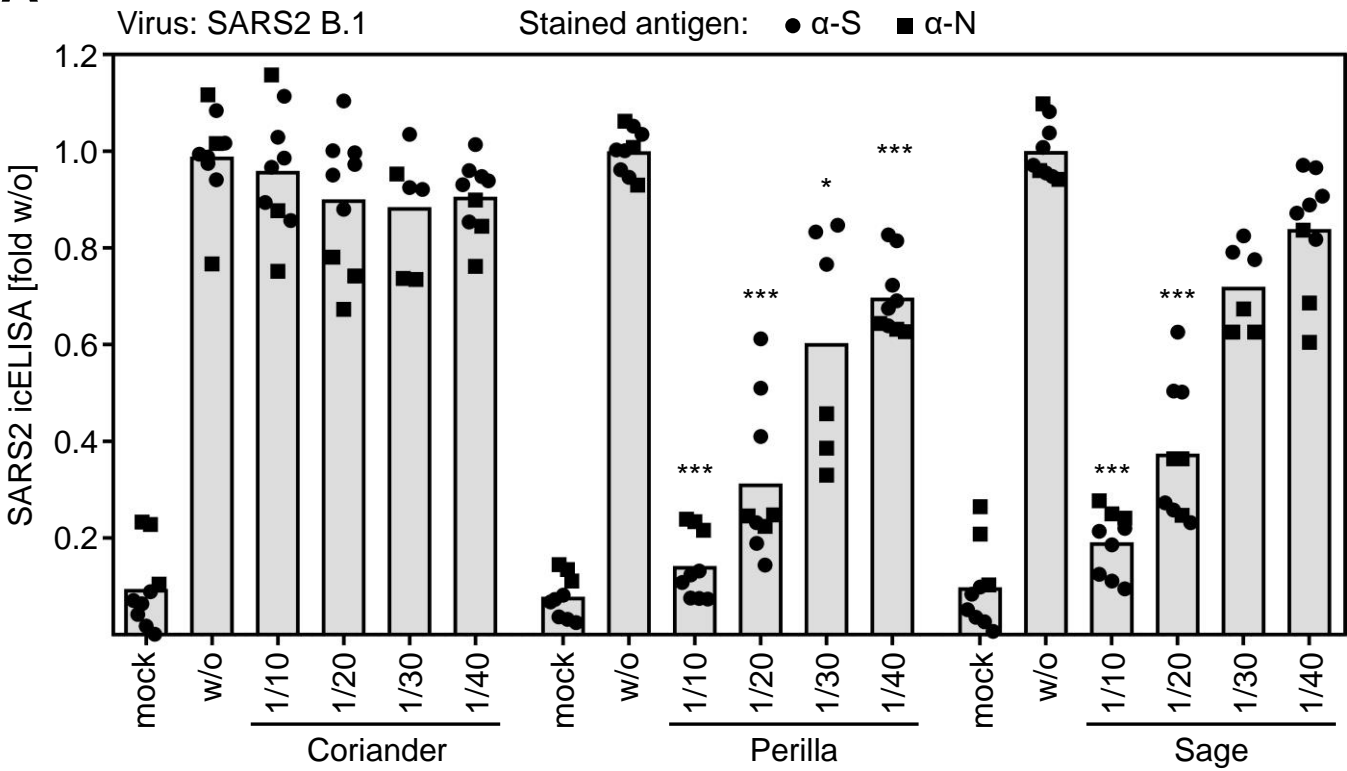

**B**

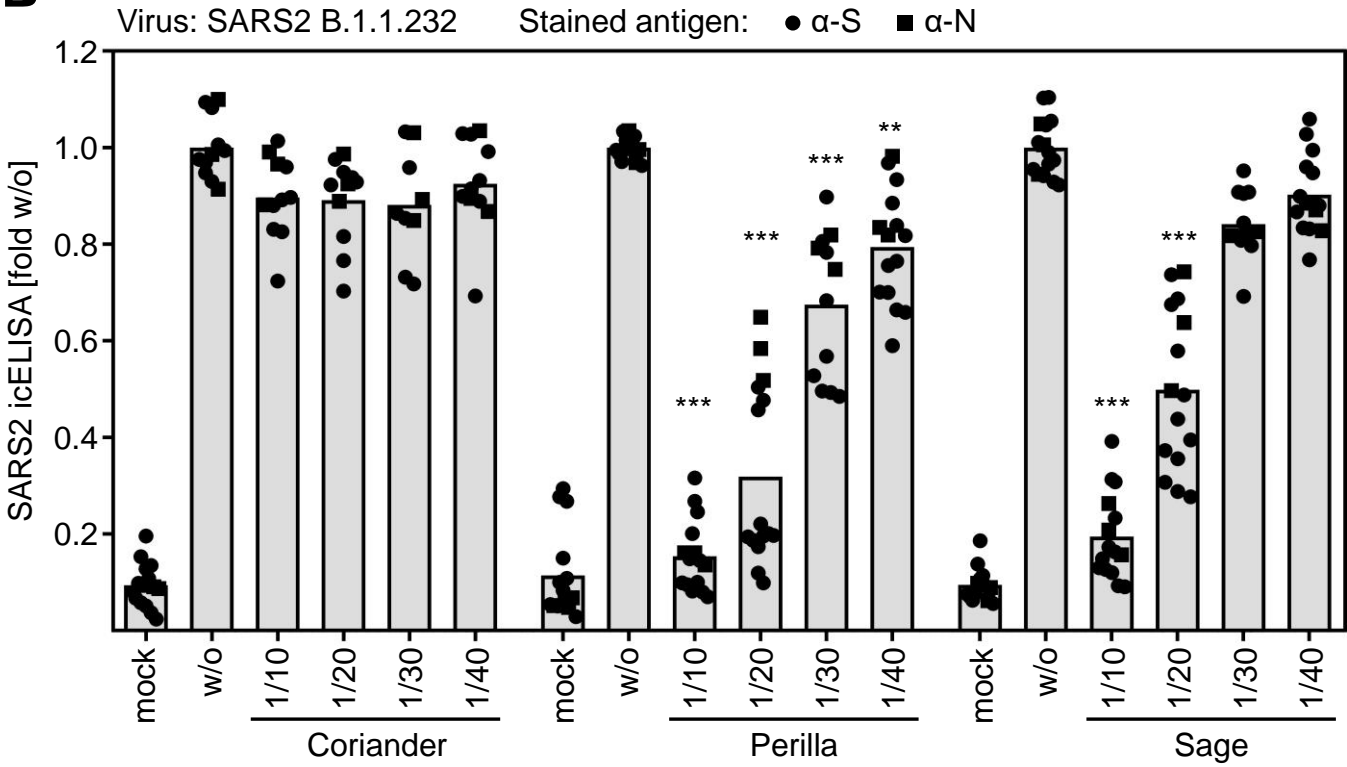

Supplement: Supplementary file 3 — Additional file 3: Fig. S2. Two different clinical SARS-CoV-2 isolates exhibit almost identical susceptibilities towards perilla and sage. A, B Pooled icELISA data of 3 (A, SARS-CoV-2 variant B.1) and 5 (B, SARS-CoV-2 variant B.1.1.232) independent experiments of SARS-CoV-2-infected Vero E6 cells after treatment with herbal infusions and icELISA using α-S or α-N mAbs for staining. Data are expressed as relative change in optical density compared to the untreated control. The perilla- and sage-treated conditions were compared to the corresponding coriander-treated condition (same dilution) by one-way ANOVA. **, p<0.01. ***, p<0.001. [file 12915_2022_1468_MOESM3_ESM.pdf]

Additional file 4: Figure S3

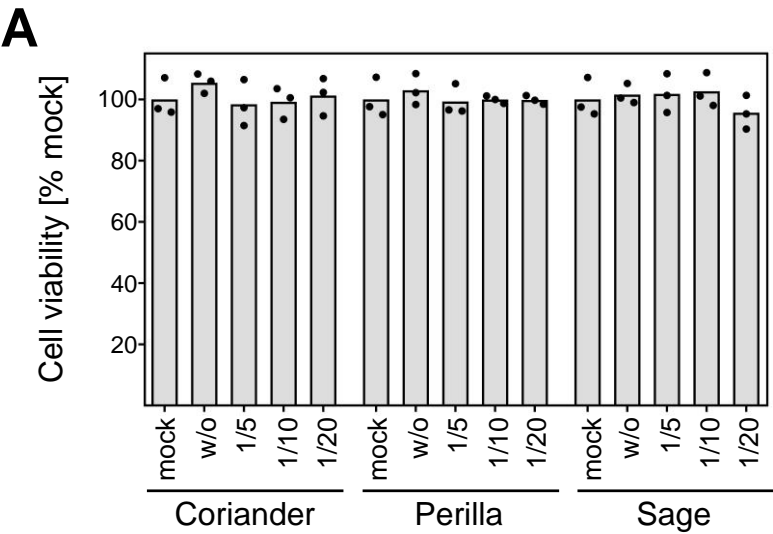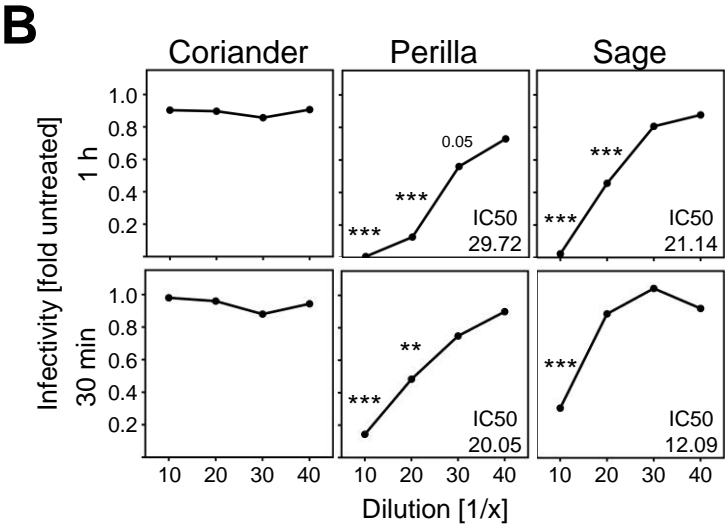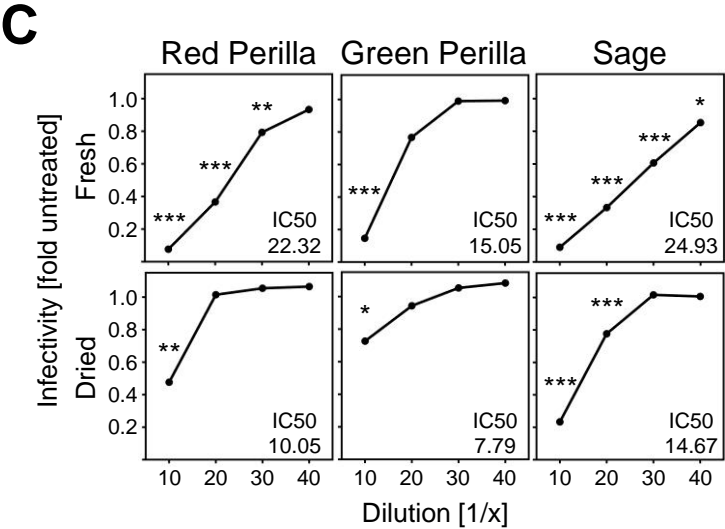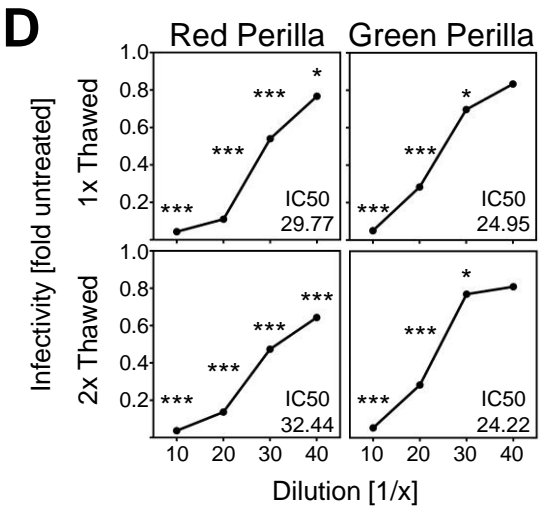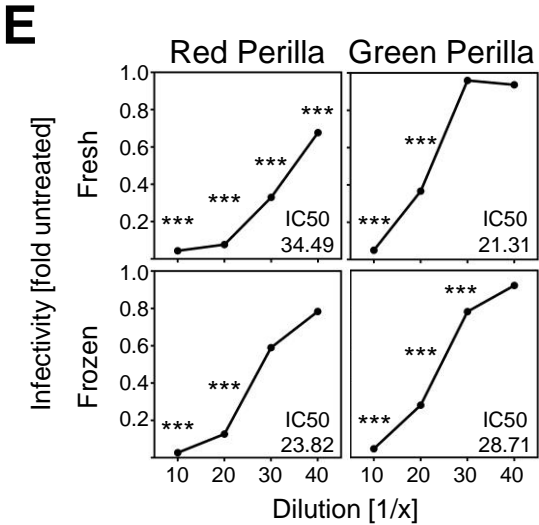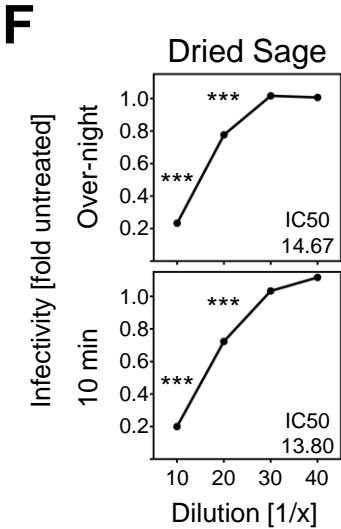

Supplement: Supplementary file 4 — Additional file 4: Fig. S3. Preserved sage and perilla leaves retain bioactive compounds. A The one-hour treatment with the herbal infusions is not cytotoxic. Vero E6 cells were treated with indicated dilutions of herbal infusions in parallel to an infection experiment. At 18 h post treatment, cell viability was analyzed by Orangu cell counting solution (Cell Guidance Systems). See Additional file 1 for individual data values. B Representative dose-response curves of SARS-CoV-2-infected Vero E6 cells (2000 PFU per well) after treatment with herbal infusions. Upper panel depicts the results for 1 h of treatment, lower panel the results for 30 min of treatment. Data are expressed as relative change in infectivity compared to the untreated control. Each condition was analyzed in triplicate. The perilla- and sage-treated conditions were compared to the corresponding coriander-treated condition (same dilution) by one-way ANOVA. **, p<0.01. ***, p<0.001. C Representative dose-response curves of SARS-CoV-2-infected Vero E6 cells after treatment with aqueous infusions generated from fresh or dried red perilla, green perilla, or sage for 1 h. Each condition was analyzed in triplicate. All conditions were compared to the untreated control by one-way ANOVA. *, p<0.05. **, p<0.01. ***, p<0.001. D Representative dose-response curves of SARS-CoV-2-infected Vero E6 cells after treatment with aliquots of herbal infusions that were thawed once or twice. Each condition was analyzed in triplicate. All conditions were compared to the untreated control by one-way ANOVA. *, p<0.05. ***, p<0.001. E Representative dose-response curves of SARS-CoV-2-infected Vero E6 cells after treatment with aqueous infusions generated from fresh or frozen red or green perilla. Each condition was analyzed in triplicate. All conditions were compared to the untreated control by one-way ANOVA. ***, p<0.001. F Representative dose-response curves of SARS-CoV-2-infected Vero E6 cells after treatment with herbal [file 12915_2022_1468_MOESM4_ESM.pdf]

Additional file 5: Figure S4

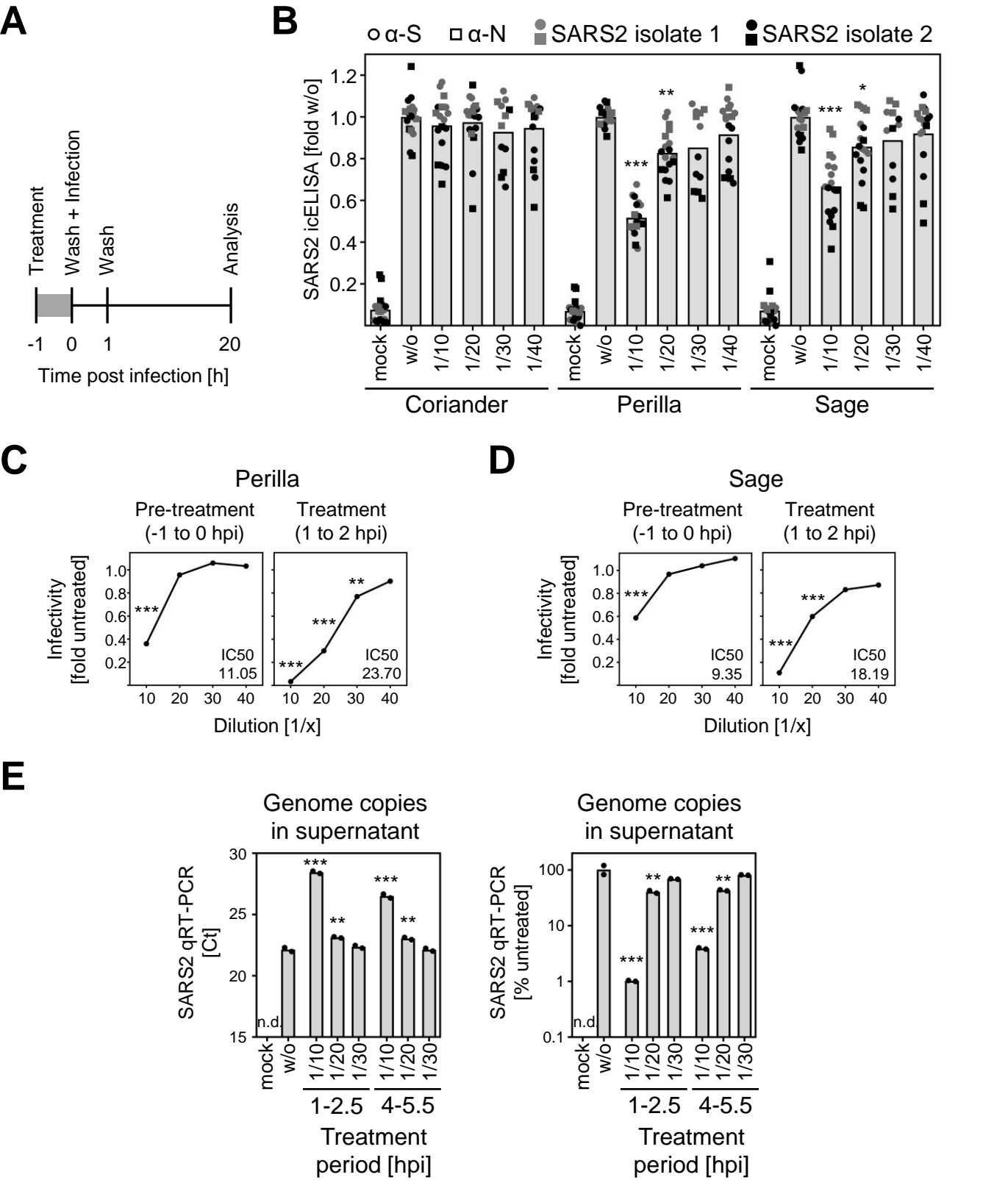

Supplement: Supplementary file 5 — Additional file 5: Fig. S4. Perilla and sage elicit in vitro antiviral activity when administered prior to infection. A Scheme of the experimental setup for the in vitro analysis of prophylactic effects against SARS-CoV-2. B Pooled icELISA data of 6 independent experiments of SARS-CoV-2-infected Vero E6 cells after pre-treatment with herbal infusions using two distinct SARS-CoV-2 isolates (B.1 and B.1.1.232) for infection of Vero E6 and α-S or α-N mAbs for staining. Data are expressed as relative change in optical density compared to the untreated control. The perilla- and sage-treated conditions were compared to the corresponding coriander-treated condition (same dilution) by one-way ANOVA. *, p<0.05. **, p<0.01. ***, p<0.001. C, D Comparison of pre-treatment and treatment (-1 to 0 h p.i. versus 1 to 2 h p.i.) of SARS-CoV-2-infected Vero E6 cells (2000 PFU per well). Each condition was analyzed in triplicate. See Additional file 1 for individual data values. All conditions were compared to the untreated control by one-way ANOVA. **, p<0.01. ***, p<0.001. E Vero E6 cells were infected with SARS-CoV-2 (2000 PFU per well). At 1 or 4 h p.i., cells were treated with perilla infusion for 1.5 h. At 20 h p.i., supernatant was collected for RNA preparation and subsequent qRT-PCR analysis. qRT-PCR data are shown in Ct value and calculated relative change in genome copies compared to the untreated control. Each condition was analyzed in duplicate. See Additional file 1 for individual data values. The perilla-treated conditions were compared to the untreated control by one-way ANOVA. *, p<0.05. **, p<0.01. ***, p<0.001. [file 12915_2022_1468_MOESM5_ESM.pdf]

**A**

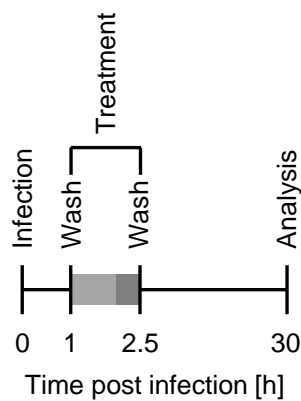

**B**

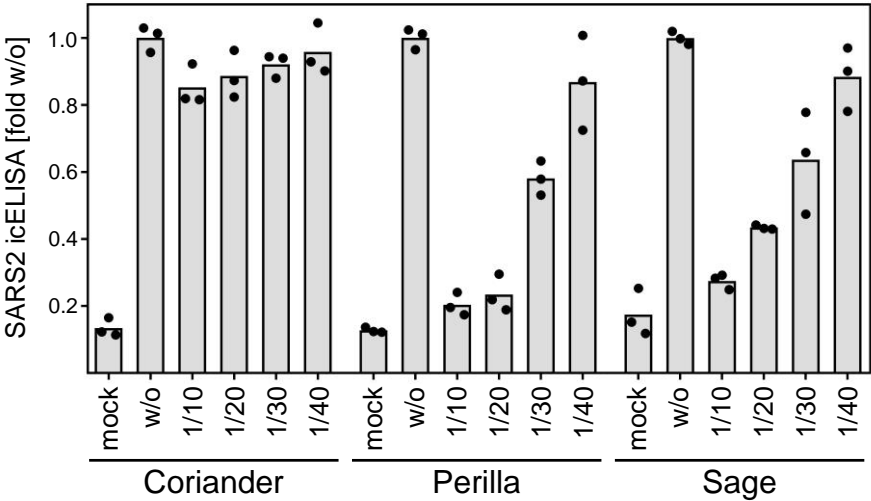

Supplement: Supplementary file 6 — Additional file 6: Fig. S5. Perilla and sage confer protection against SARS-CoV-2 infection in human cells. A Scheme of the applied experimental setup. B icELISA data (α-S staining) of SARS-CoV-2-infected Caco-2 cells (2000 PFU per well). Data are expressed as relative change in optical density compared to the untreated control. Each condition was analyzed in triplicate. See Additional file 1 for individual data values. [file 12915_2022_1468_MOESM6_ESM.pdf]

**A**

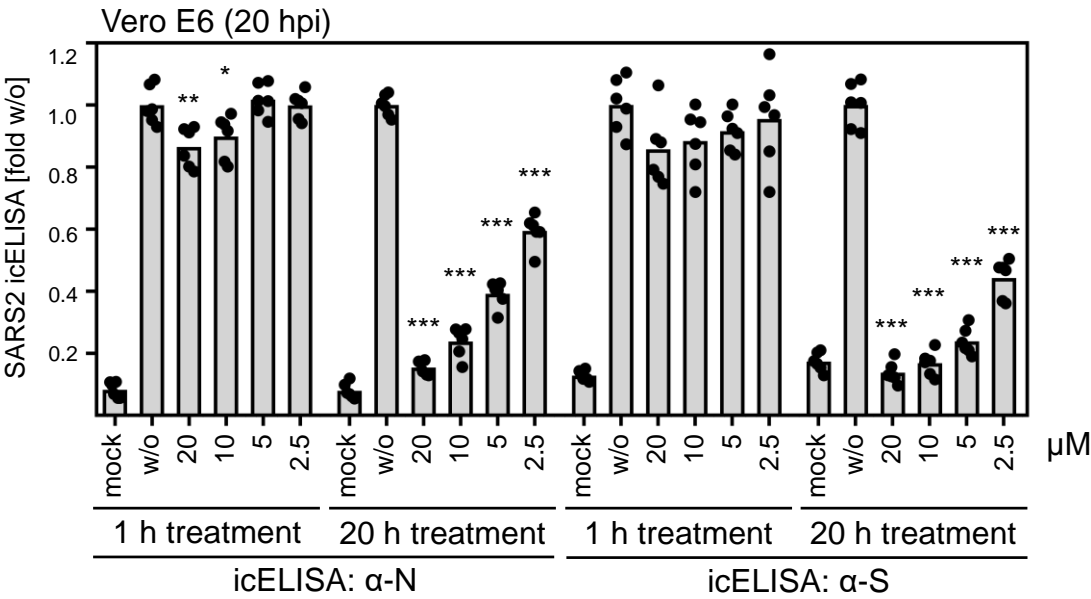

**B**

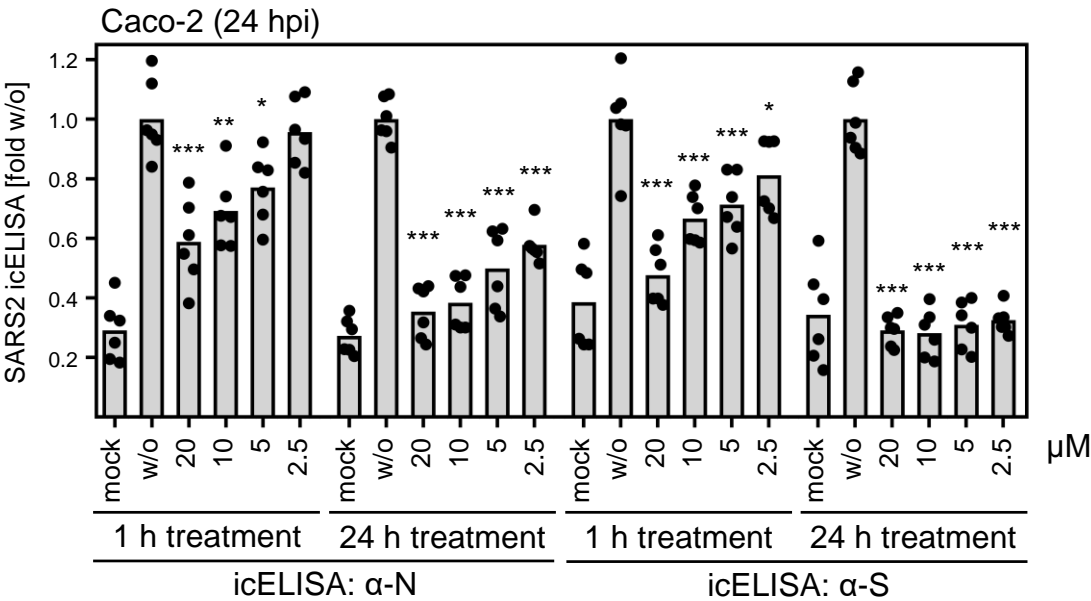

Supplement: Supplementary file 7 — Additional file 7: Fig. S6. Longer treatment periods enhances remdesivir-mediated protection against SARS-CoV-2 infection. A, B Pooled icELISA data of 2 independent experiments of SARS-CoV-2-infected Vero E6 (A) and Caco-2 (B) cells after treatment with remdesivir at 1 h p.i for 1 h p.i. or for the complete time period before fixation. Cells were fixed at 20 or 24 h p.i. and stained with α-S (Vero E6) or α-N mAb (Caco-2). Each condition was analyzed in triplicate. The treated conditions were compared to the untreated control by one-way ANOVA. *, p<0.05. **, p<0.01. ***, p<0.001. [file 12915_2022_1468_MOESM7_ESM.pdf]

A

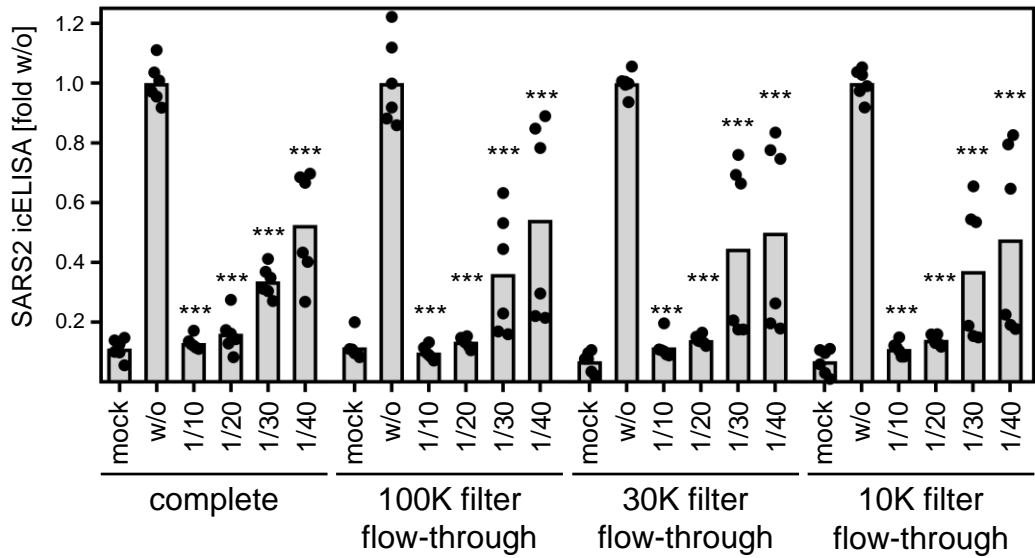

B

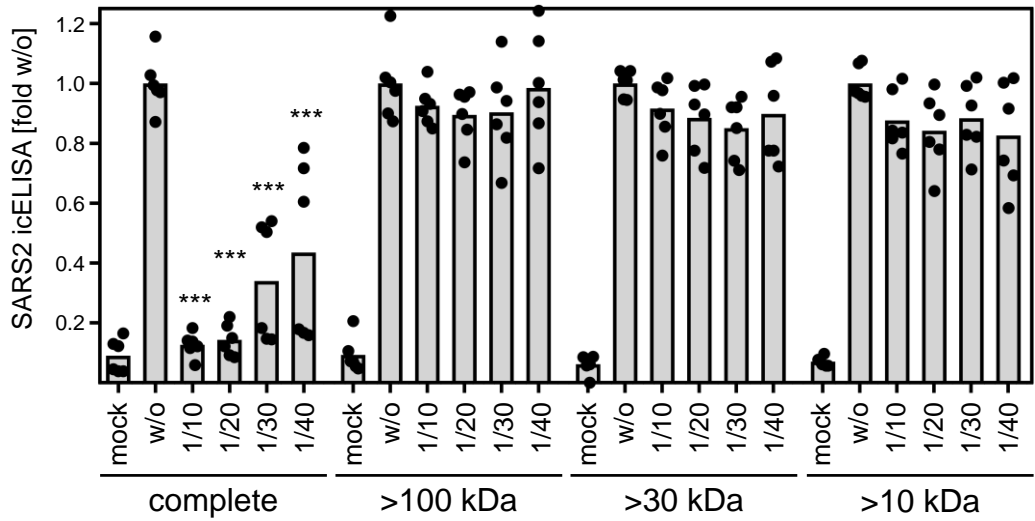

Supplement: Supplementary file 8 — Additional file 8: Fig. S7. Size exclusion and protein fractionation analyses of perilla infusion disclosed compounds <10 kDa as active components. Pooled icELISA data (α-S staining) of 2 independent experiments of SARS-CoV-2-infected Vero E6 cells after treatment with indicated fractions of perilla infusion. Data are expressed as relative change in optical density compared to the untreated control. Protein fractionation of perilla infusion components was conducted by use of Amicon 100K, 30K, and 10K filters. Each condition was analyzed in triplicate. See Additional file 1 for individual data values. Dilutions and concentrations by the fractionation steps were considered. All conditions were compared to the untreated control by one-way ANOVA. ***, p<0.001. A Perilla flow-through of Amicon 100K, 30K, and 10K filters were analyzed. B Perilla fractions >100 kDa, >30 kDa, and >10 kDa were analyzed. [file 12915_2022_1468_MOESM8_ESM.pdf]

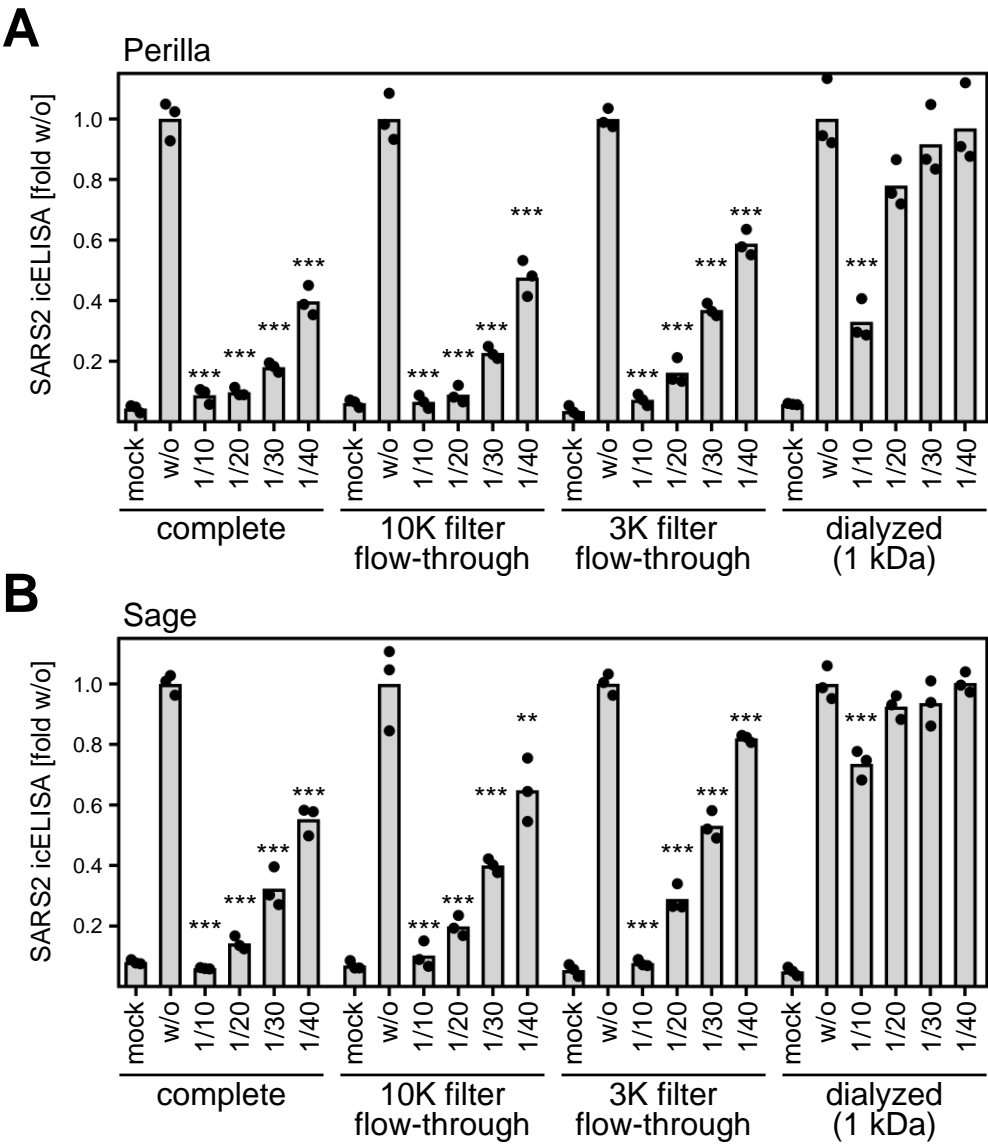

Supplement: Supplementary file 9 — Additional file 9: Fig. S8. Size exclusion and protein fractionation analyses of perilla and sage infusions disclosed compounds <3 kDa as active components. A, B icELISA data of SARS-CoV-2-infected Vero E6 cells after treatment with indicated fractions of herbal infusions (A, perilla; B, sage) at 1 h p.i. for 1 h. Cells were fixed at 20 h p.i. and stained with α-S mAb. Data are expressed as relative change in optical density compared to the untreated control. Each condition was analyzed in triplicate. See Additional file 1 for individual data values. Protein fractionation of herbal infusion components was conducted by use of Amicon 10K and 3K filters. The fraction of proteins >1 kDa was obtained by dialysis. Dilutions and concentrations by the fractionation steps were considered. The treated conditions were compared to the untreated control by one-way ANOVA. **, p<0.01. ***, p<0.001. [file 12915_2022_1468_MOESM9_ESM.pdf]

Additional file 10: Figure S9

**A**

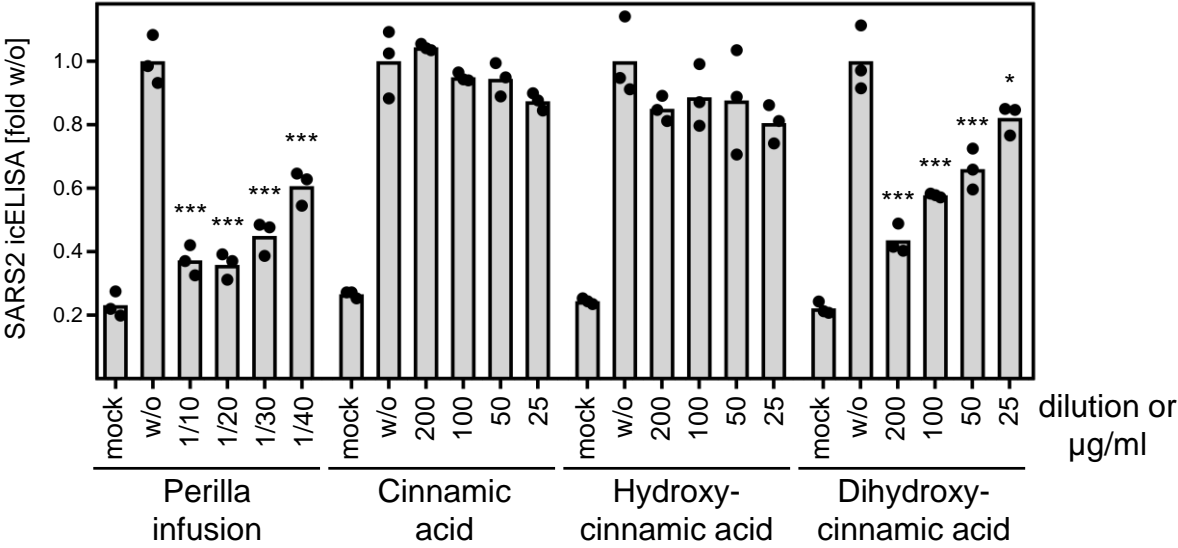

**B**

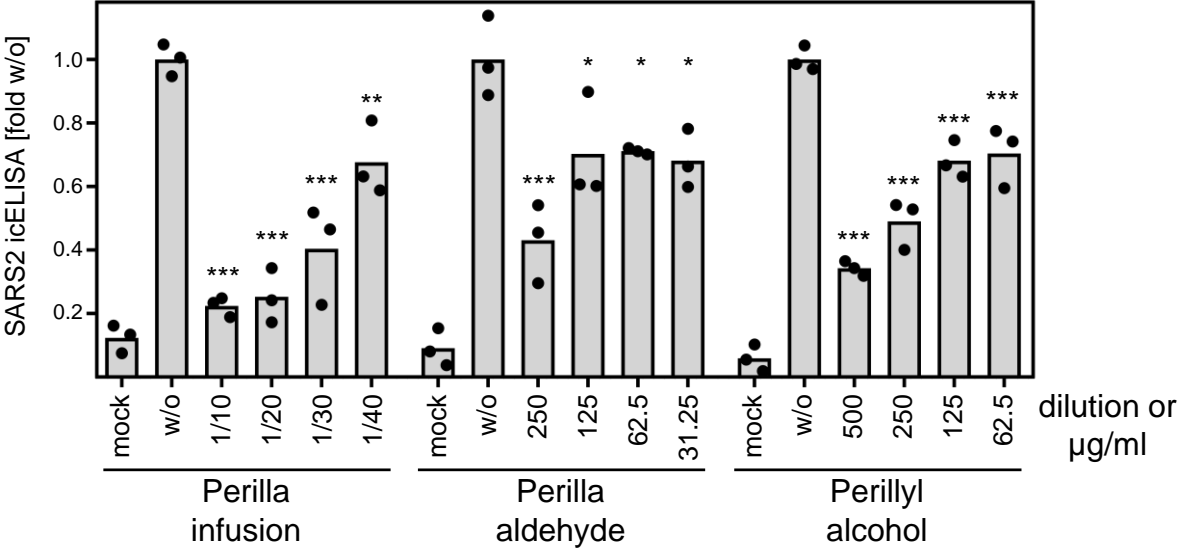

Supplement: Supplementary file 10 — Additional file 10: Fig. S9. Dihydroxy-cinnamic acid, perilla aldehyde, and perillyl alcohol are antiviral components of herbal infusions. A, B icELISA data of SARS-CoV-2-infected Caco-2 cells after treatment with indicated substances at 1.5 h p.i. for 1.5 h. Data are expressed as relative change in optical density compared to the untreated control. Each condition was analyzed in triplicate. All treated conditions were compared to the untreated control by one-way ANOVA. *, p<0.05. ***, p<0.001. [file 12915_2022_1468_MOESM10_ESM.pdf]

A

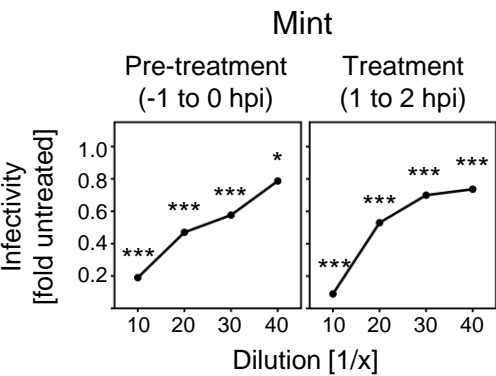

B

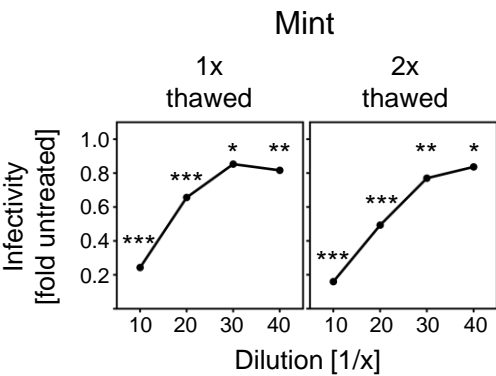

Supplement: Supplementary file 11 — Additional file 11: Fig. S10. Treatment with mint infusions elicit antiviral activity in vitro. A Comparison of pre-treatment and treatment (-1 to 0 h p.i. versus 1 to 2 h p.i.) of SARS-CoV-2-infected Vero E6 cells (2000 PFU per well). Each condition was analyzed in triplicate. See Additional file 1 for individual data values. All conditions were compared to the untreated control by one-way ANOVA. *, p<0.05. **, p<0.01. ***, p<0.001. B Representative dose-response curves of SARS-CoV-2-infected Vero E6 cells after treatment with aliquots of herbal infusions that were thawed once or twice. Each condition was analyzed in triplicate. See Additional file 1 for individual data values. All conditions were compared to the untreated control by one-way ANOVA. *, p<0.05. ***, p<0.001. [file 12915_2022_1468_MOESM11_ESM.pdf]

Additional file 14: Figure S11

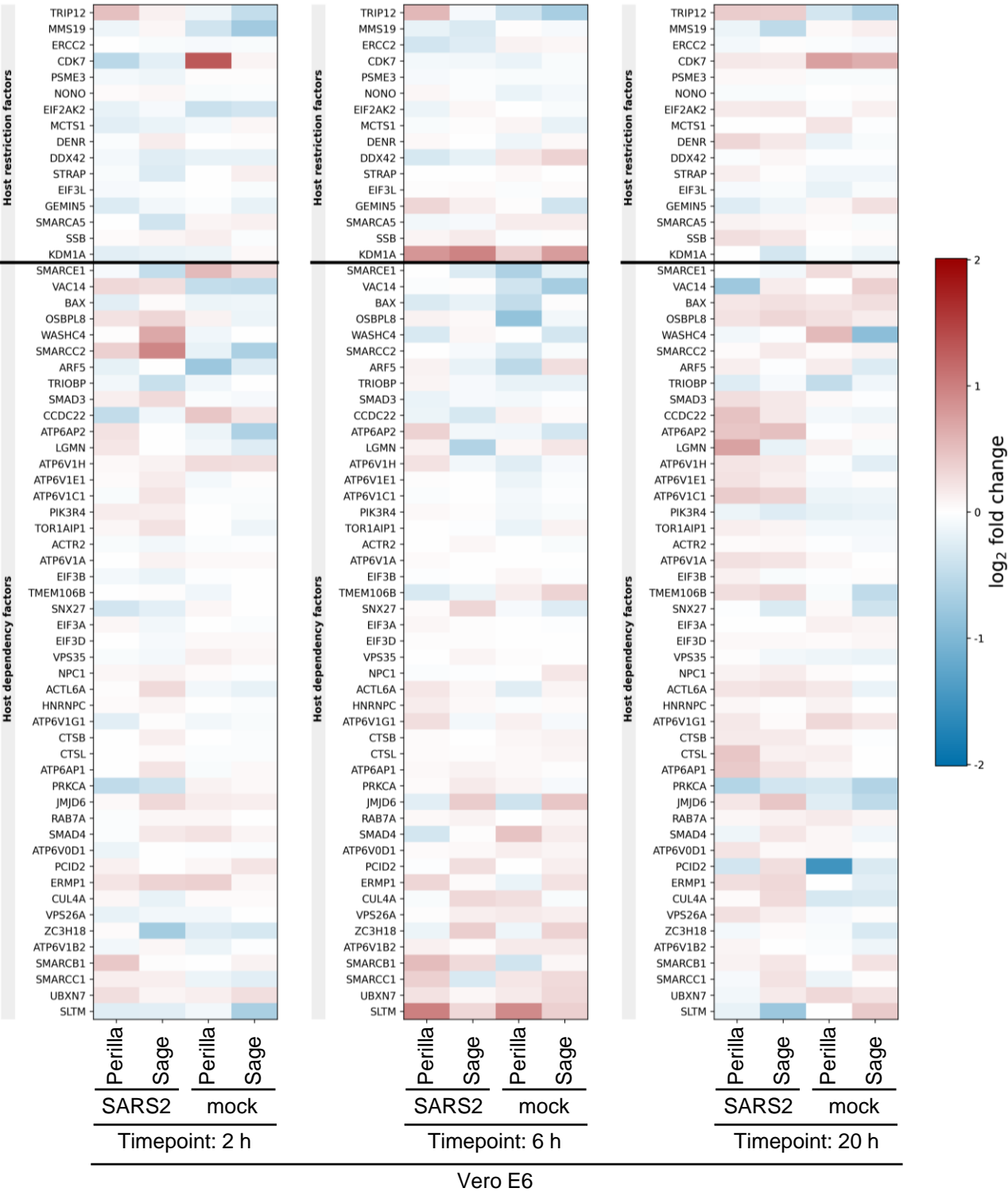

Supplement: Supplementary file 14 — Additional file 14: Fig. S11. Herbal infusion-induced changes of proposed host restriction and host dependency factors in Vero E6 cells. SARS-CoV-2-infected Vero E6 cells as well as mock controls were treated at 1 h p.i. with perilla and sage infusion for 1 h. At 2, 6, and 20 h p.i., cells were lysed and subjected to global MS analysis. Each condition was analyzed in quintuplicate. Proposed HRFs and HDFs [34, 35, 36],which were consistently quantified across all investigated conditions, were analyzed regarding the changes induced by treatment with perilla or sage infusions. The ratio of the treated condition to the respective untreated condition is shown as log2fold change. [file 12915_2022_1468_MOESM14_ESM.pdf]

Additional file 15: Figure S12

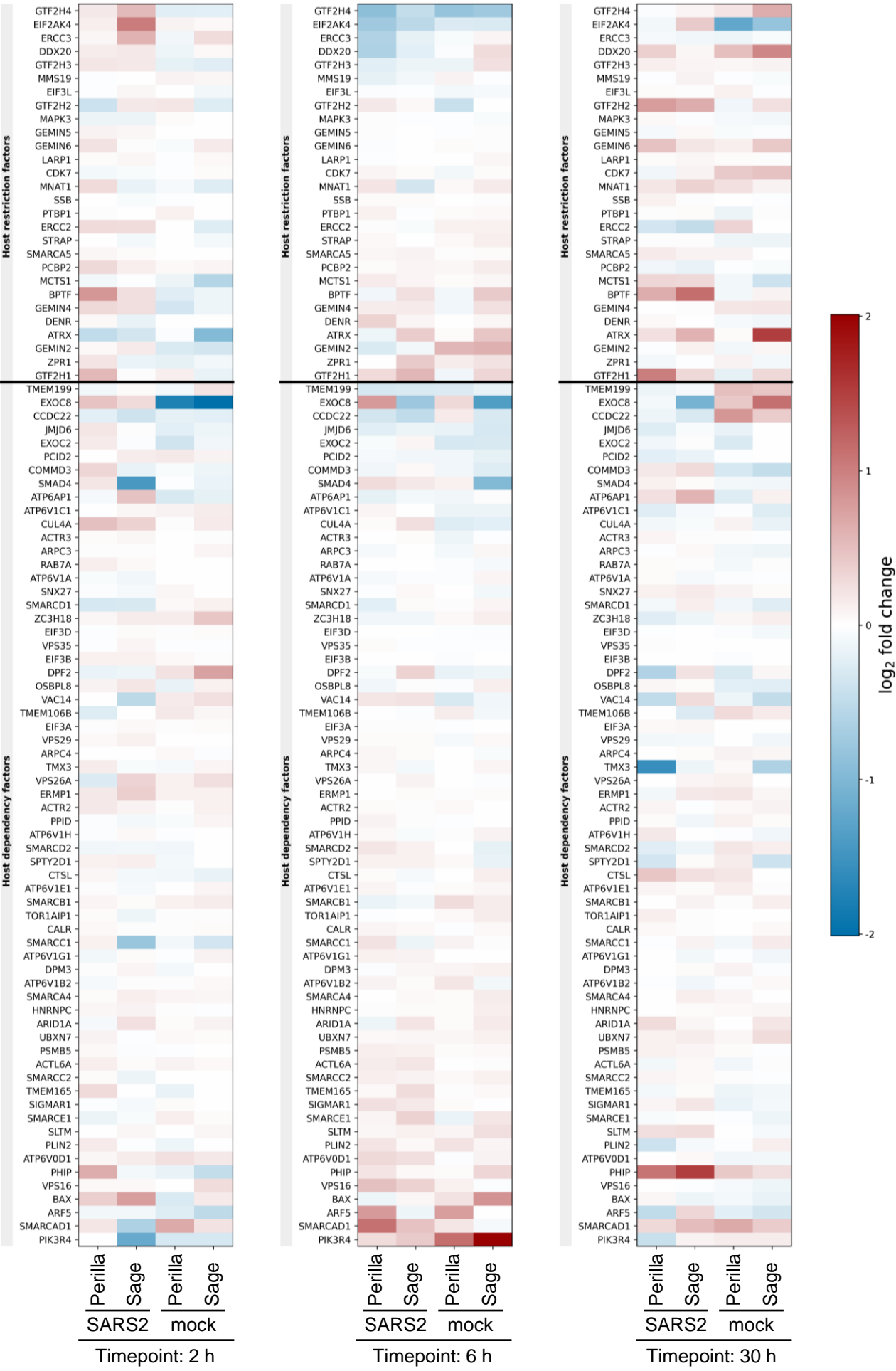

Supplement: Supplementary file 15 — Additional file 15: Fig. S12. Herbal infusion-induced changes of proposed host restriction and host dependency factors in Caco-2 cells. SARS-CoV-2-infected Caco-2 cells as well as mock controls were treated at 1 h p.i. with perilla and sage infusion for 1 h. At 2, 6, and 30 h p.i., cells were lysed and subjected to global MS analysis. Each condition was analyzed in quadruplicate or quintuplicate. Proposed HRFs and HDFs [34, 35, 36],which were consistently quantified across all investigated conditions, were analyzed regarding the changes induced by treatment with perilla or sage infusions. The ratio of the treated condition to the respective untreated condition is shown as log2fold change. [file 12915_2022_1468_MOESM15_ESM.pdf]

Additional file 16: Figure S13

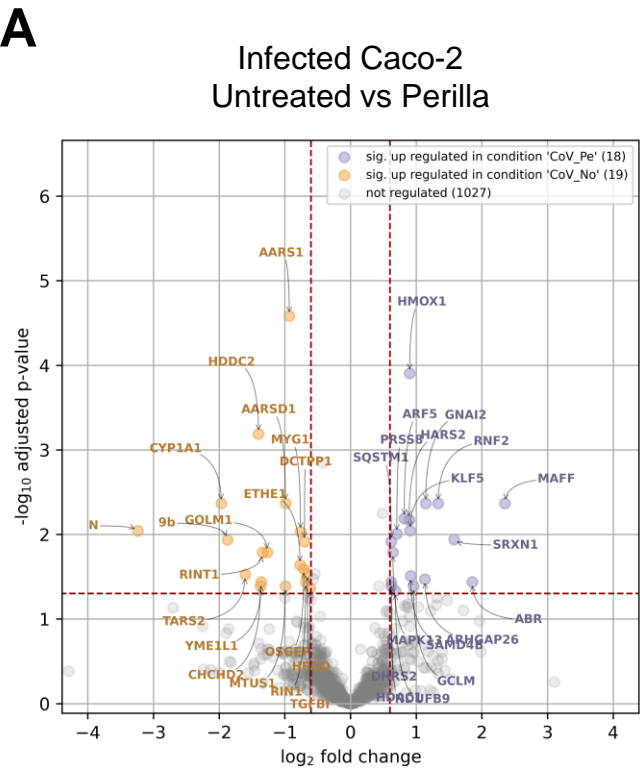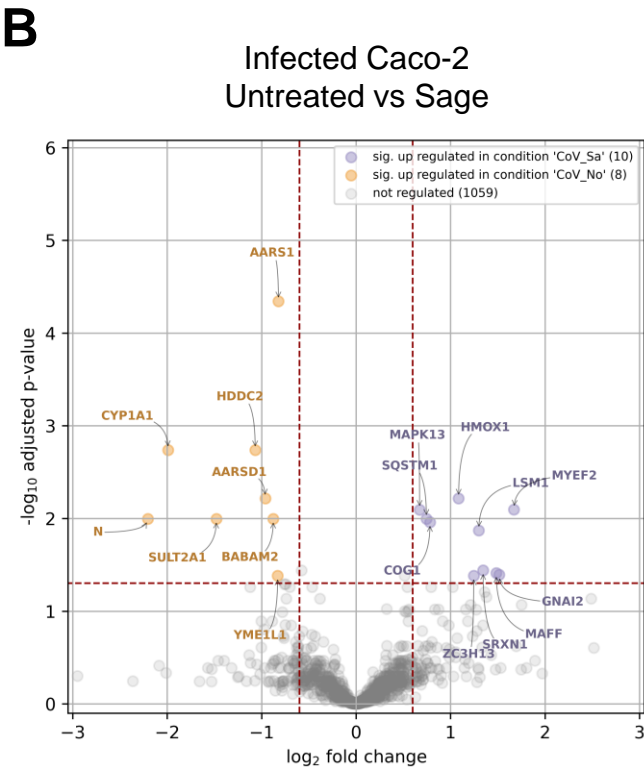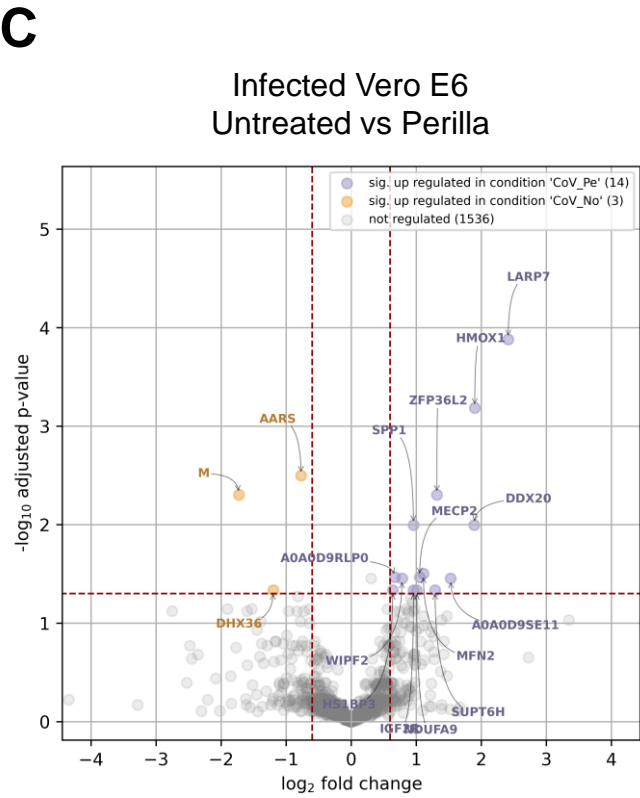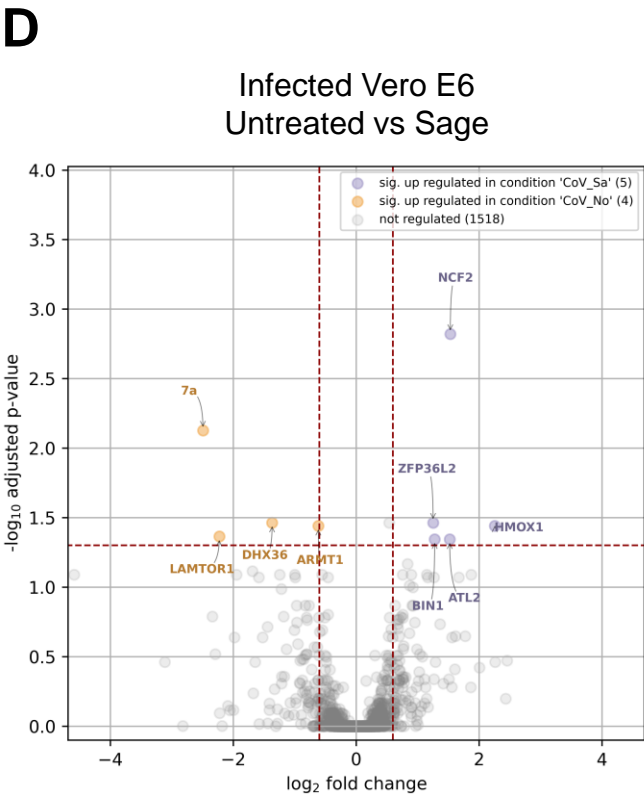

Supplement: Supplementary file 16 — Additional file 16: Fig. S13. Perilla and sage infusion-induced changes at 6 h p.i. in infected Caco-2 and Vero E6 cells. A-D Volcano plots of MS results obtained from infected Caco-2 (A, B) or Vero E6 (C, D) at 6 h p.i. Proteome Discoverer normalized data were filtered (at least two unique peptides per protein group required) and proteins with a coefficient of variation <20.0% over all measurements (also across treatments) were removed (see “Methods” section for details). [file 12915_2022_1468_MOESM16_ESM.pdf]

Additional file 17: Figure S14

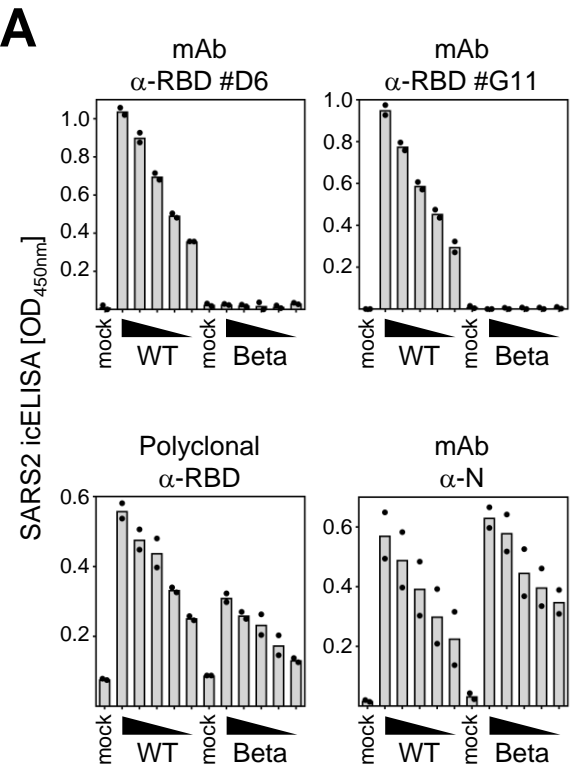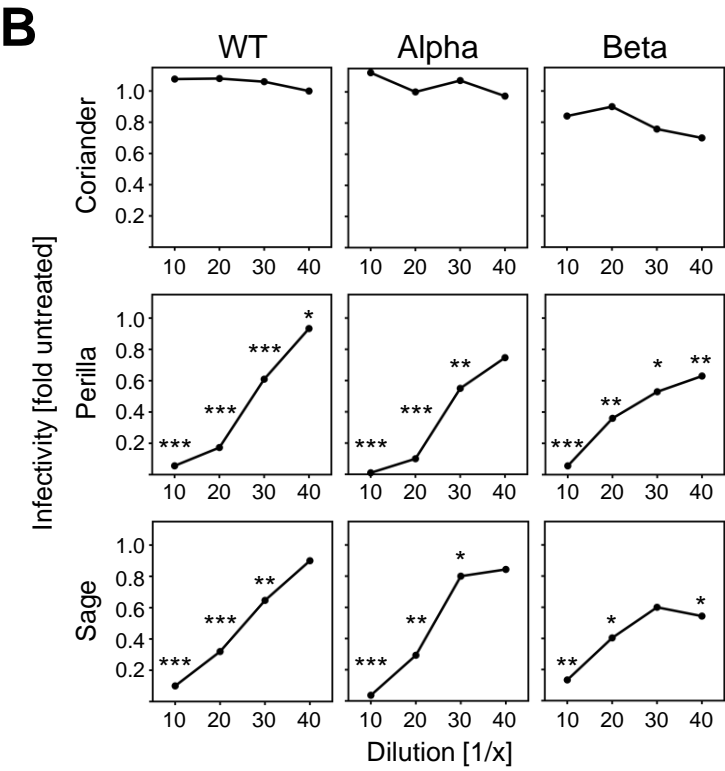

Supplement: Supplementary file 17 — Additional file 17: Fig. S14. Herbal infusions exhibit antiviral activity against SARS-CoV-2 variants of concern. A Vero E6 cells were infected with graded doses of SARS-CoV-2 wt and Beta (B.1.351). At 20 h p.i., cells were fixed and analyzed by icELISA using different antibodies recognizing the receptor-binding domain of Spike (RBD) or the nucleocapsid protein (N). See Additional file 1 for individual data values. B Representative dose-response curves of Vero E6 cells infected with SARS-CoV-2 wt and variants of concern (Alpha/B.1.1.7 and Beta/B.1.351) after treatment with aqueous infusions of coriander, perilla, or sage at 1 h p.i. for 1 h. SARS-CoV-2 replication was analyzed at 20 h p.i. by icELISA (α-N staining). Data are expressed as relative change in infectivity compared to the untreated control. Each condition was analyzed in triplicate. See Additional file 1 for individual data values. The comparison of the herb-treated samples of SARS-CoV-2 to the untreated control by one-way ANOVA showed for all dilutions of coriander no significance. The perilla- and sage-treated conditions of SARS-CoV-2 were also compared to the corresponding coriander-treated condition (same dilution) and these results are depicted in the diagram. *, p<0.05. **, p<0.01. ***, p<0.001. [file 12915_2022_1468_MOESM17_ESM.pdf]
